# Enhanced Carrier Collection in Cd/In-Based Dual Buffers in Kesterite Thin-Film Solar Cells from Nanoparticle Inks

**DOI:** 10.1021/acsaem.3c01622

**Published:** 2023-10-27

**Authors:** Stephen Campbell, Guillaume Zoppi, Leon Bowen, Pietro Maiello, Vincent Barrioz, Neil S. Beattie, Yongtao Qu

**Affiliations:** † Department of Mathematics, Physics and Electrical Engineering, Northumbria University, Newcastle-upon-Tyne NE1 8ST, United Kingdom; ‡ Department of Physics, Durham University, Durham DH1 3LE, United Kingdom

**Keywords:** kesterite, CZTSSe, nanoparticle inks, In_2_S_3_−CdS buffer, EBIC

## Abstract

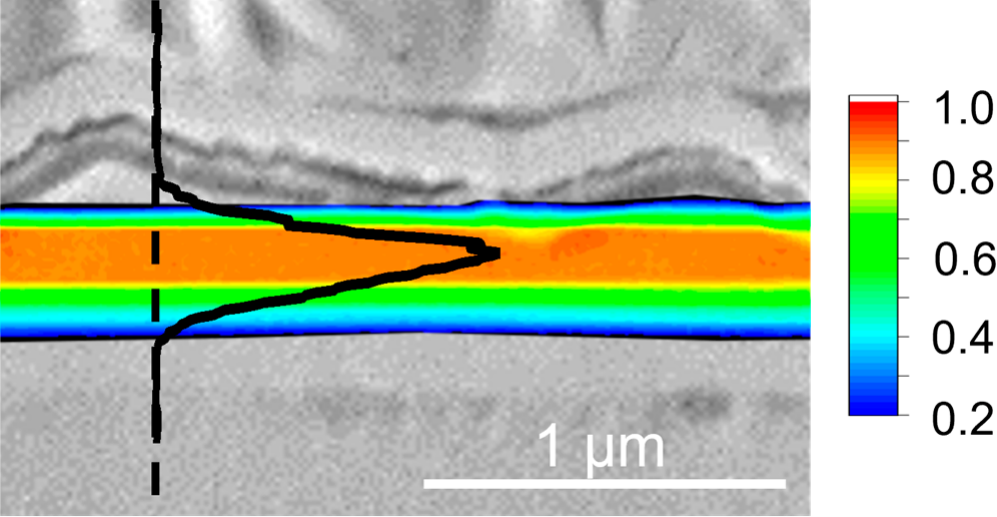

Increasing the power conversion efficiency (PCE) of kesterite Cu_2_ZnSn(S,Se)_4_ (CZTSSe) solar cells has remained challenging over the past decade, in part due to open-circuit voltage (*V*_OC_)-limiting defect states at the absorber/buffer interface. Previously, we found that substituting the conventional CdS buffer layer with In_2_S_3_ in CZTSSe devices fabricated from nanoparticle inks produced an increase in the apparent doping density of the CZTSSe film and a higher built-in voltage arising from a more favorable energy-band alignment at the absorber/buffer interface. However, any associated gain in *V*_OC_ was negated by the introduction of photoactive defects at the interface. This present study incorporates a hybrid Cd/In dual buffer in CZTSSe devices that demonstrate an average relative increase of 11.5% in PCE compared to CZTSSe devices with a standard CdS buffer. Current density–voltage analysis using a double-diode model revealed the presence of (i) a large recombination current in the quasi-neutral region (QNR) of the CZTSSe absorber in the standard CdS-based device, (ii) a large recombination current in the space-charge region (SCR) of the hybrid buffer CZTSSe–In_2_S_3_–CdS device, and (iii) reduced recombination currents in both the QNR and SCR of the CZTSSe–CdS–In_2_S_3_ device. This accounts for a notable 9.0% average increase in the short-circuit current density (*J*_SC_) observed in CZTSSe–CdS–In_2_S_3_ in comparison to the CdS-only CZTSSe solar cells. Energy-dispersive X-ray, secondary-ion mass spectroscopy, and grazing-incidence X-ray diffraction compositional analysis of the CZTSSe layer in the three types of kesterite solar cells suggest that there is diffusion of elemental In and Cd into the absorbers with a hybrid buffer. Enhanced Cd diffusion concomitant with a double postdeposition heat treatment of the hybrid buffer layers in the CZTSSe–CdS–In_2_S_3_ device increases carrier collection and extraction and boosts *J*_SC_. This is evidenced by electron-beam-induced current measurements, where higher current generation and collection near to the p–n junction is observed, accounting for the increase in *J*_SC_ in this device. It is expected that optimization of the heat treatment of the hybrid buffer layers will lead to further improvements in the device performance.

## Introduction

Among all renewable energy sources (wind, water, solar, etc.), photovoltaic (PV) technology is the most promising way to harvest ambient light energy silently and unobtrusively into electricity, committing to the net-zero greenhouse emission strategy.^1^ Closely related to the prominent thin-film technology copper–indium–gallium selenide but with increased Earth abundance of the constituent elements, kesterite emerged as one of the most promising PV absorber materials because of its low cost and excellent and stable optoelectronic properties.^2,3^ To date, Cu_2_ZnSn(S,Se)_4_ (CZTSSe) thin-film solar cells (TFSCs) demonstrated promising power conversion efficiencies (PCEs) of 13.8% (certified) and 14.9% (reported) at the laboratory scale,^4−6^ while the theoretically predicted efficiency for kesterites is over 32%, which gives large motivation and window to further enhance the device performance. Importantly, kesterite solar cells made from nanoparticle inks have the potential to provide disruptively high specific power solar modules on flexible substrates that are ideal for integration to “self-powered” distributed Internet of Things (IoT) applications.^7−11^

While TFSCs fabricated from a kesterite absorber provide an Earth-abundant and stable energy-harvesting solution, their commercialization has been historically restricted, in part due to their *V*_OC_-limiting defect states at both the absorber bulk and absorber/buffer interface.^12,13^ It was found that substituting the conventional CdS buffer layer with In_2_S_3_ in kesterite devices produced an increase in the apparent doping density of the CZTSSe film and a higher built-in voltage arising from a more favorable energy-band alignment at the absorber/buffer interface.^14−16^ However, any associated gain in *V*_OC_ was negated by the introduction of photoactive defects at the interface, as reported in our previous work.^17^ It is believed that elemental doping due to interdiffusion at absorber/buffer heterojunctions plays an important role in passivating interface defects and determining the kesterite solar cells’ performance.^18,19^

In this study, therefore, a CdS/In_2_S_3_ hybrid buffer structure was explored in CZTSSe devices to understand how the buffer structure will influence the elemental diffusion at the heterojunction and determine the device performance. Spectral response measurements of hybrid buffer devices confirmed the presence of photoactive interface defects when the In_2_S_3_ buffer is adjacent to the CZTSSe absorber. Current density–voltage analysis using a double-diode model revealed the presence of (i) a large recombination current in the quasi-neutral region (QNR) of the CZTSSe absorber in the standard CdS-based device, (ii) a large recombination current in the space-charge region (SCR) of the hybrid buffer CZTSSe–In_2_S_3_–CdS device, and (iii) reduced recombination currents in both the QNR and SCR of the CZTSSe–CdS–In_2_S_3_ device. Further measurement including energy-dispersive X-ray (EDX), secondary-ion mass spectroscopy (SIMS), and grazing-incidence X-ray diffraction (GIXRD) compositional analysis of the CZTSSe layer in the three types of kesterite solar cells suggests that there is diffusion of elemental In into the absorbers with a hybrid buffer. We found that a middle CdS layer between the CZTSSe absorber and top In_2_S_3_ buffer is essential to provide a proper doping density of the SCR region of CZTSSe without adversely affecting the overall device performance. Hereafter, devices with structures CZTSSe–CdS, CZTSSe–In_2_S_3_–CdS, and CZTSSe–CdS–In_2_S_3_ will be referred to as devices H, D, and E, respectively.

## Results and Discussion

### Device Characteristics

**Figure 1:**
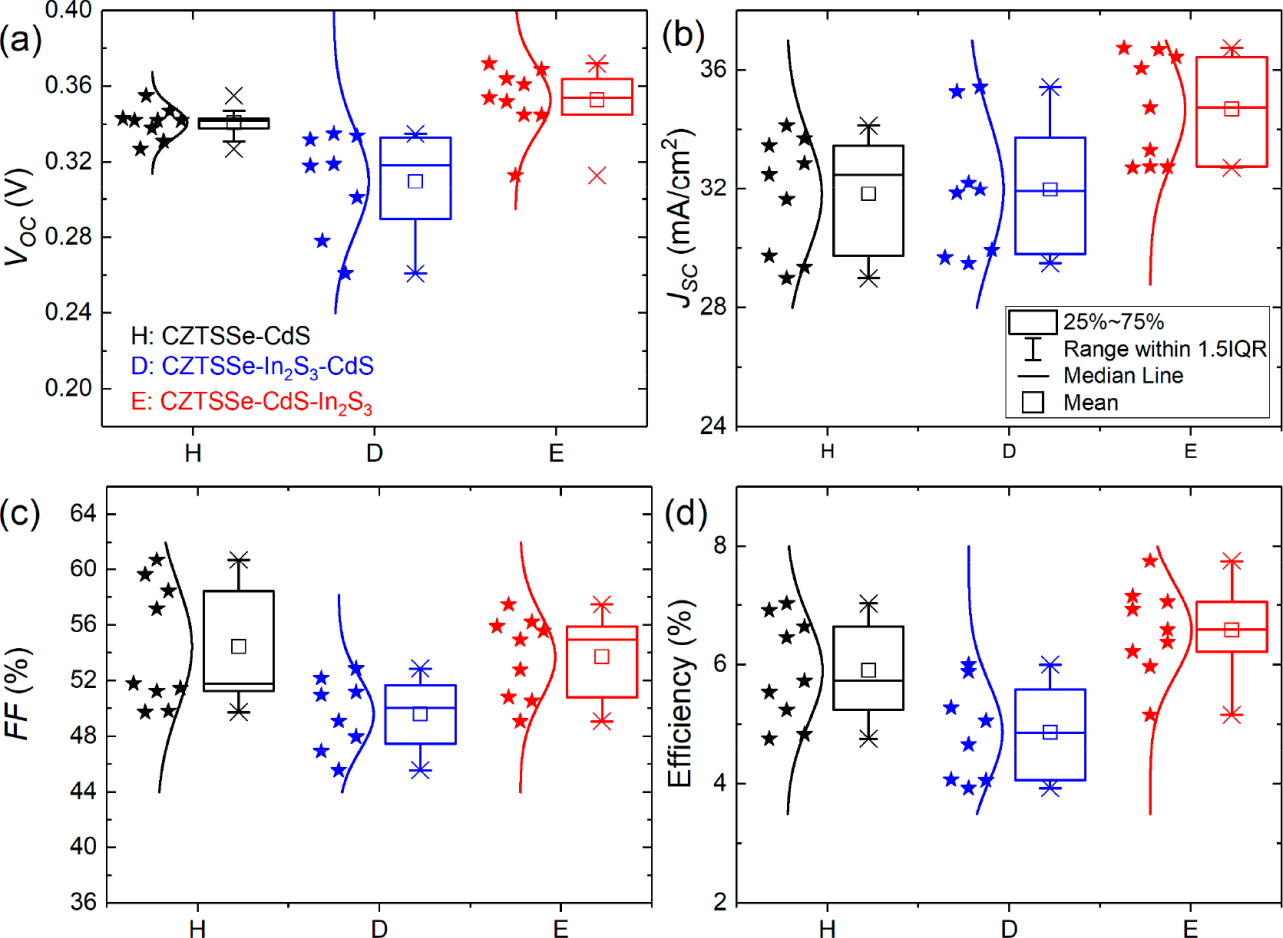
Box plots of the distribution of (a) *V*_OC_, (b) *J*_SC_, (c) FF, and (d) efficiency of all nine cells for each device structure. The □ symbol is the average value, and the × symbol represents minimum and maximum values. The three horizontal lines of each box represent 25%, 50%, and 75% of the data distribution. The whisker range is determined by the standard deviation of the data.

**Figure 2:**
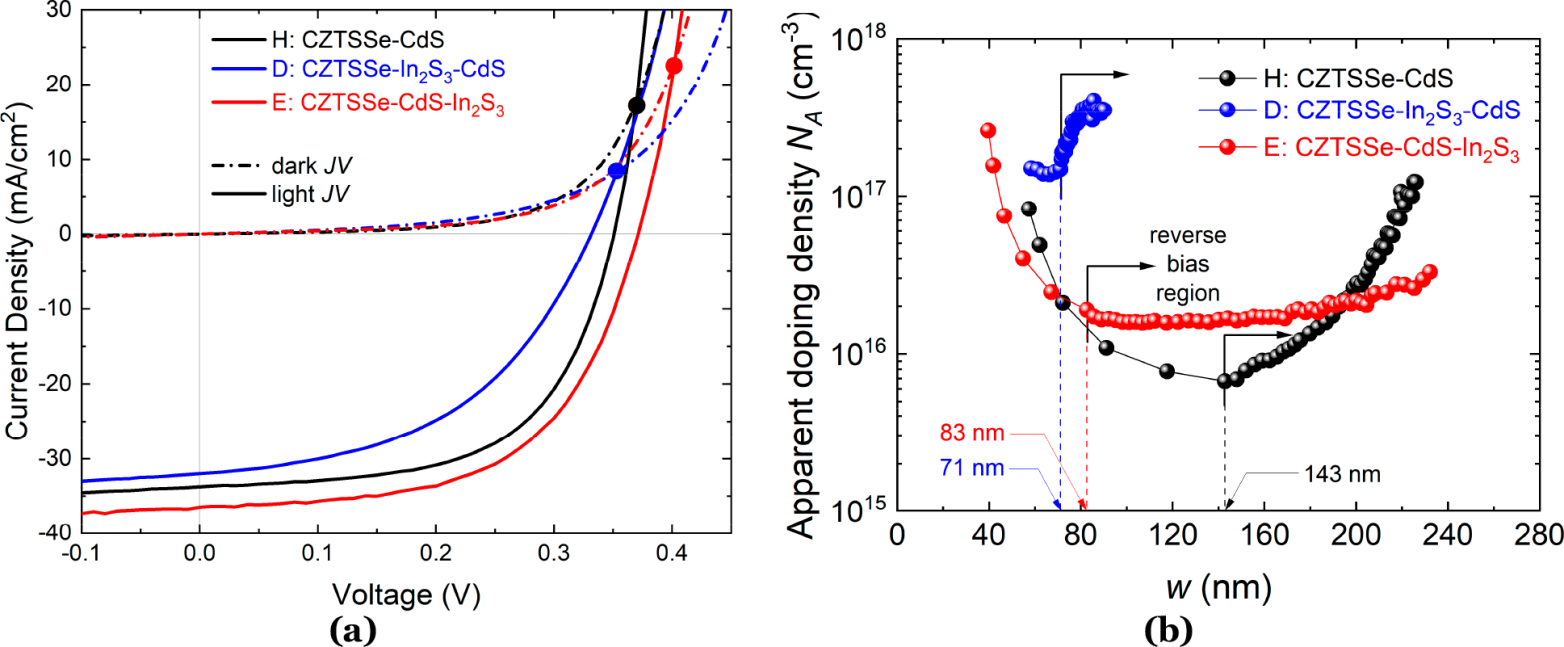
(a) Light (solid lines) and dark (dash-dotted lines) *J*–*V* curves for all champion device types. Solid circles mark the crossover points between light and dark *J*–*V* plots. (b) *C*–*V* depth profiles with indicated *w*_SCR_ values at zero bias.

Figure 1 shows the distribution of device parameters in the CZTSSe solar cells with different buffers, with Table 1 showing the average parameter values for a set of nine solar cells (champion device values in parentheses). Evident is the increase in the open-circuit voltage (*V*_OC_), short-circuit current density (*J*_SC_), and PCE (η) of device E compared to the other devices. Device D generally performed worse than the other two devices. Both device sets with a dual buffer exhibit a fill factor (FF) lower than that of the standard devices with a single CdS buffer. The decrease in FF for devices with a dual buffer layer can be directly related to an increase in the series resistance (*R*_S_) for those devices. The typical *R*_S_ value for the dual buffer cells studied is ∼1.9 Ω cm^2^ compared to ∼1.0 Ω cm^2^ for the reference CdS-buffered cells, while the shunt resistance (*R*_SH_) was similar in all device types (∼140 Ω cm^2^). Higher *R*_S_ in the dual buffer devices can be attributed to an increase in the combined thickness of the CdS/In_2_S_3_ layers (see the Electron Microscopy and Composition section).^20^ Most notable is the average increase in *J*_SC_ of type E solar cells (34.7 mA/cm^2^) compared to those of types H (31.8 mA/cm^2^) and D (32.0 mA/cm^2^), with champion device E achieving a *J*_SC_ of 36.7 mA/cm^2^. Subsequently, this solar cell achieved a PCE of 7.75%. The *J*–*V* curves of champion solar cells for all device types measured in the dark and under 1-sun illumination are plotted in Figure 2a.

**Table 1:** Average Device Parameters for the CZTSSe Solar Cells, with the Best-Performing Device Values in Parentheses

device type	*V*_OC_ (mV)	*J*_SC_ (mA/cm^2^)	FF (%)	efficiency (%)
H: CZTSSe–CdS	341 (355)	31.8 (34.1)	54.5 (60.7)	5.90 (7.03)
D: CZTSSe–In_2_S_3_–CdS	310 (335)	32.0 (35.4)	49.6 (52.9)	4.87 (6.00)
E: CZTSSe–CdS–In_2_S_3_	353 (372)	34.7 (36.7)	53.7 (57.5)	6.58 (7.75)

The dark and illuminated *J*–*V* curves exhibit crossover behavior, which is indicative of an electrical barrier either in the buffer/absorber interface or at the absorber/Mo metal contact due to a thick interfacial layer of MoSe_2_.^21−23^ Because all devices fabricated for this study were made from the same batch of CZTS nanoparticle inks and subjected to the same selenization conditions, it is unlikely that a thick MoSe_2_ layer is the cause of the low crossover point. Our previous studies have shown that CZTSSe devices fabricated from CZTS nanoparticle inks typically have a low hole barrier of ∼40 meV at the back-contact, suggesting that the back-contact is not the predominant factor influencing carrier extraction.^19,24^ This suggests that a current-blocking barrier exists at the buffer/absorber interface in all types of devices, with a higher barrier present when In_2_S_3_ is deposited directly on top of the CZTSSe absorber (indicated by the lower *J*–*V* crossover point in device D). The dark *J*–*V* curves in Figure 2a were fitted using the double-diode model for an n^+^–p device (Figure S1), described by
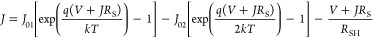
1where *k* is the Boltzmann constant, *T* is the temperature, *R*_S_ is the series resistance, *R*_SH_ is the shunt resistance, and *J*_01_ and *J*_02_ are the reverse saturation currents relating to the recombination currents in the QNR and SCR of the solar cell, respectively (Table 2). Practical fitting of the illuminated *J*–*V* curves is difficult because small fluctuations in the light intensity overwhelm the effects of the second diode, which relates to the SCR. It is apparent that device E has a lower recombination current in the SCR (*J*_02_ = 6.9 × 10^–3^ mA/cm^2^) compared to the other device types (H, *J*_02_ = 4.6 × 10^–2^ mA/cm^2^; D, *J*_02_ = 6.4 × 10^–1^ mA/cm^2^), which is concomitant with higher *J*_SC_ observed in type E devices. Conversely, device D shows a lower recombination current in the bulk of the CZTSSe absorber, where *J*_01_ = 1.6 × 10^–6^ mA/cm^2^ in contrast to *J*_01_ values of 2.6 × 10^–4^ and 7.3 × 10^–5^ mA/cm^2^ for devices H and E, respectively (Table 2). Explanations for this behavior will be explored in the Results and Discussion section. The performance of kesterite solar cells can be limited by heterojunction interface defects and deep-level defects in the SCR.^25−27^

**Table 2:** Recombination Current Values (*J*_01_ and *J*_02_) of the Best-Performing Cells for Each Device Type Determined by Double-Diode Analysis (According to Equation 1) with the Apparent Doping Density (*N*_A_) and SCR Width (*w*_SCR_) of the Corresponding Devices

device type	*J*_01_ QNR (mA/cm^2^)	*J*_02_ SCR (mA/cm^2^)	*w*_SCR_ (nm)	*N*_A_ (cm^–3^)
H: CZTSSe–CdS	2.6 × 10^–4^	4.6 × 10^–2^	143	8.9 × 10^15^
D: CZTSSe–In_2_S_3_–CdS	1.6 × 10^–6^	6.4 × 10^–1^	71	1.7 × 10^17^
E: CZTSSe–CdS–In_2_S_3_	7.2 × 10^–5^	6.9 × 10^–3^	83	1.6 × 10^16^

Capacitance–voltage (*C*–*V*) profiling measurements were performed to determine the apparent doping density (*N*_A_), SCR width (*w*_SCR_), and built-in potential (*V*_BI_) of all device types. When the bias voltage is set to *V* = 0, *N*_A_ and *V*_BI_ can be determined from Mott–Schottky analysis of the *C*–*V* data, which is expressed by *N*_A_ = −2(d*C*^–2^/d*V*)/*q*ε_0_ε_r_, where *C* is the capacitance/cell area, ε_0_ is the permittivity of free space, and ε_r_ is the dielectric constant of CZTSSe (Figure S2). As such, built-in voltages of 0.182, 0.841, and 0.126 V were determined for devices H, D, and E, respectively. The relatively high *V*_BI_ value observed in device D is likely due to a positive “spike-like” conduction band offset at the CZTSSe/In_2_S_3_ interface, which was demonstrated in our previous study of CZTSSe solar cells with a In_2_S_3_ buffer.^17^

Devices with a large *V*_BI_ should have a correspondingly high *V*_OC_, which is not the case for devices with the structure CZTSSe–In_2_S_3_–CdS and could be related to increased levels of defects at the buffer/absorber interface. Additionally, *N*_A_ values of 8.4 × 10^15^, 1.8 × 10^17^, and 1.7 × 10^16^ cm^–3^ were determined for devices H, D, and E, respectively (Table 2).

**Figure 3:**
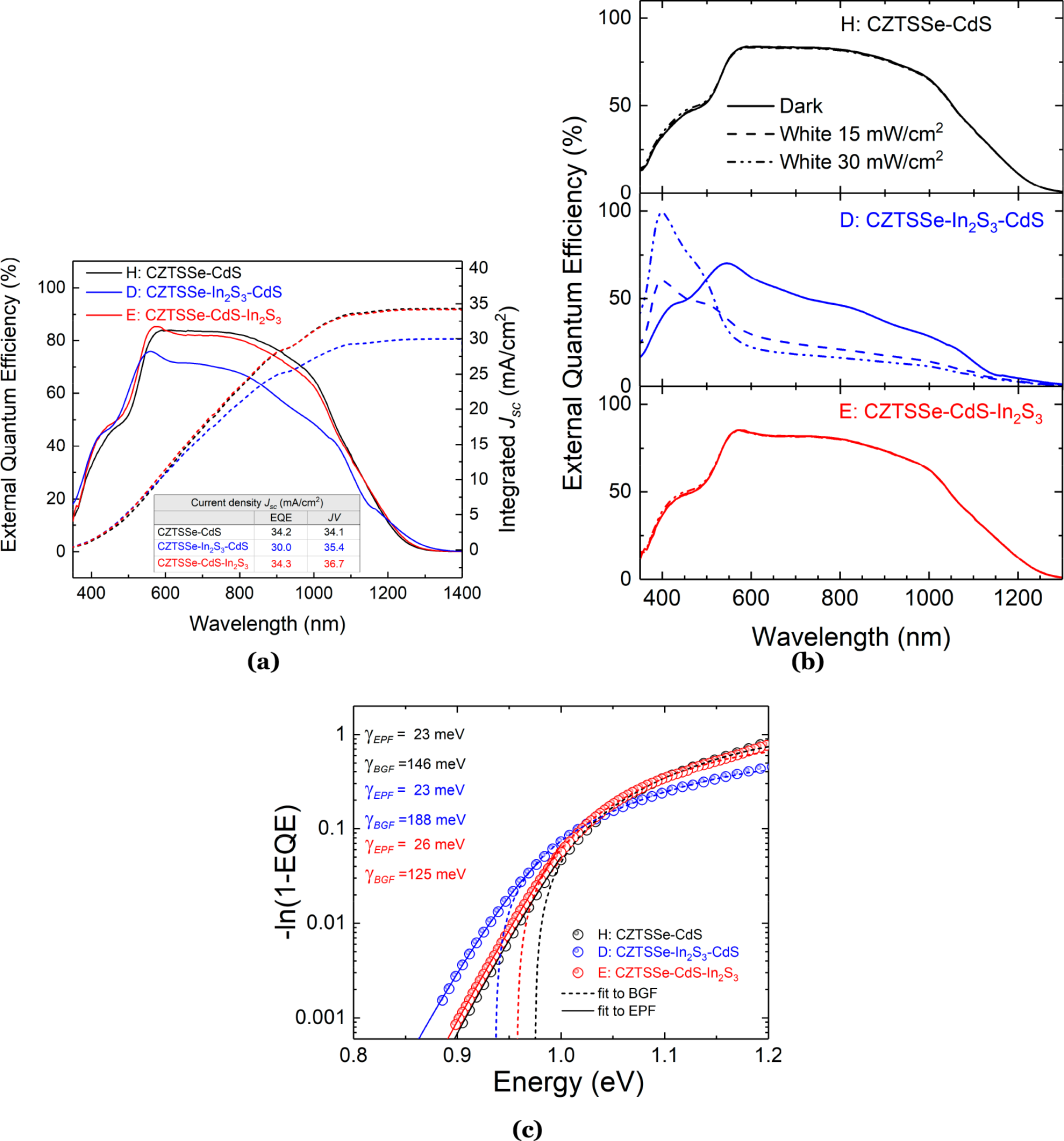
(a) EQE spectra with integrated *J*_SC_ of the best cells with an area of 0.16 cm^2^. (b) EQE with white-light bias (at 15 and 30 mW/cm^2^) and without light bias (dark) for different device types. (c) Fitting of the absorption coefficient α [α ln(1 – EQE)] below the band gap of each device to estimate the magnitude of electrostatic potential fluctuations (γ_EPF_) and band-gap fluctuations (γ_BGF_).

The notable increase in the doping density for device D has previously been observed in kesterite solar cells, which employ In_2_S_3_ as a buffer layer and was ascribed to significant In diffusion into the absorber as a result of device processing conditions.^14,15,18^ Elemental diffusion into the CZTSSe absorber will be addressed in detail in the Results and Discussion section. Figure 2b shows the apparent doping density profile at a distance *w* from the p–n junction width. Devices H and D show a “U”-shaped depth-dependent doping profile often seen in thin-film chalcopyrite and kesterite solar cells, with a minimum doping concentration typically in the range of a few 10^15^ cm^–3^ for moderate applied voltage bias with significantly higher doping density at high forward and reverse bias.^17,28−31^ An increase in the apparent doping density toward increasing forward bias has been attributed to minority carrier injection and parasitic resistances and the increase with higher reverse bias related to the presence of deep defects.^32^ Werner et al. have suggested that the SCR capacitance in TFSCs containing Cd- or Zn-incorporated buffers follows the model of a linearly graded junction and is a result of elemental intermixing at the buffer/absorber interface, which alters the apparent doping concentration.^28^ However, device E shows an extended flat region in the depth-dependent doping profile, suggesting a more uniform doping concentration that extends from the region near the buffer/absorber interface into the bulk of the CZTSSe absorber. The SCR width (*w*_SCR_) is calculated to be 143, 71, and 83 nm for devices H, D, and E, respectively. Both device types with a dual buffer exhibit a significant narrowing of the SCR compared to the standard device. This can be directly attributed to the higher doping density observed in the devices with a dual buffer because the SCR width ratio of n-to-p-type semiconductors in a p–n junction is equal to the ratio of apparent acceptor-to-donor density, i.e., *w*_SCR,p_/*w*_SCR,n_, where *w*_SCR,p_ and *w*_SCR,n_ are the SCR widths in p- and n-type semiconductors, respectively, and *N*_D_ and *N*_A_ are the apparent donor and acceptor densities, respectively (Table 2). From Table 2, it appears that the apparent doping density of the CZTSSe absorbers in the studied devices is linked to the recombination current in the SCR of their respective devices. To gain deeper insight into the carrier collection efficiency, external quantum efficiency (EQE) measurements were performed on all device types, with the results shown in Figure 3a. The spectra showed similar responses, whereby a steep rise in current collection is observed in the ultraviolet-blue region (<450 nm), followed by steady collection in the region of 600–850 nm and a gradual decrease at wavelengths of >850 nm. Both types of dual buffer devices showed increased current extraction at wavelengths of >550 nm due to the presence of a slightly thinner CdS layer and higher transmittance in the In_2_S_3_ layer.^17^ The most notable difference in the EQE spectra is the drop in response of device D over wavelengths of >550 nm, which could be linked to a poor minority carrier diffusion length coupled with a narrow *w*_SCR_. Because effective charge separation occurs in the depletion region in the absorber material of a solar cell, such a small *w*_SCR_ due to a high hole concentration in CZTSSe adversely affects the carrier collection. The overall lower collection efficiency in this device would also suggest a higher barrier to minority carrier (electron) transport, which is in agreement with the lower *J*–*V* crossover for this device seen in Figure 2a. A similar behavior has been observed in other kesterite solar cells that employ a In_2_S_3_ buffer.^14,33,34^ By definition, EQE represents the ratio between the numbers of generated charge carriers to the number of incident photons; therefore, *J*_SC_ can be estimated by integrating the EQE over the entire spectrum. The comparative results between *J*_SC_ extracted from *J*–*V* and EQE analysis are shown as a table inset in Figure 3a. It is evident that devices with a dual buffer layer have lower *J*_SC_ when calculated from EQE spectra. Similar results have been observed previously and were attributed to the photoactive nature of the In_2_S_3_ layer and the CZTSSe/In_2_S_3_ interface, which can influence defects and interface recombination.^14,35^ The band gaps *E*_G_ of the CZTSSe absorbers in each type of device were determined from EQE measurements (Figure S3), yielding values of 1.143, 1.187, and 1.143 eV for devices H, D, and E, respectively. Xiao et al. studied the effects of In doping on CZTS and CZTSe solar cells and found that an increasing In/Sn ratio (or In content) caused a monotonic increase in the absorber band gap.^36^ Therefore, In diffusion from the In_2_S_3_ buffer into the CZTSSe film due to In_2_S_3_/CdS film deposition/heat treatment could account for the increased band gap seen in device D.

Additionally, light-biased EQE measurements were done to study the effects of white-light illumination (at 15 and 30 mW/cm^2^) on absorption in the CZTSSe films of each device type (Figure 3b). There is little discernible difference between the unbiased and biased EQE spectra for devices H and E, while the light-biased EQE response of device D is higher in the blue part of the spectrum (wavelengths of <500 nm) and significantly lower over the remaining spectrum (wavelengths of >500 nm). This phenomenon has also been previously observed in kesterite CZTS solar cells with a CdS and/or In_2_S_3_ buffer layer,^14,35^ which was ascribed to saturated and unsaturated photoactive defects in the CdS and In_2_S_3_ layers, respectively. Under white-light illumination, these photoactive defects shrink the width of the SCR by the optical injection of red photons, and the effect is more pronounced in the device with an In_2_S_3_ buffer directly on top of the CZTSSe absorber, due to the unsaturated nature of the defects present in this film.

In a nonideal semiconductor with high defect densities, band tail states allow the absorption of photons with sub-band-gap energies. The sub-band-gap absorption in CZTSSe can be modeled in several ways: (i) Urbach tail states; (ii) band-gap fluctuations (BGF); (iii) electrostatic potential fluctuations (EPF).^17,37^ It is evident from the EQE spectra in Figure 3a that there is significant absorption of sub-band-gap photons in all device types, and further analysis is required to determine the cause. It is possible to quantify the origins of band tailing utilizing the relationship between the absorption coefficient (α) of a semiconductor and EQE. EQE is proportional to α such that α ∝ – ln[1 – EQE(*hν*)]^38^ and depends on the density of states in the fluctuating potentials of the conduction and valence energy bands. Regarding band-gap fluctuations (γ_BGF_), the model assumes a Gaussian distribution of band-gap energies centered at *E*_G,mean_ and characterized by a standard deviation σ where σ ≡ γ_BGF_:^39,40^
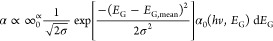
2

Shklovskii and Efros also related α to the mean amplitude of electrostatic potential fluctuations (γ_EPF_) due to a random distribution of charged defects, namely,^41^
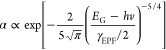
3Therefore, the above absorption models can be applied to a plot of α ∝ −ln[1 – EQE(*h*ν)] versus *h*ν (Figure 3c). It is apparent that the application of a single absorption model to the data does not adequately describe the observed behavior. In this instance, a combination of BGF and EPF models provides the best data fit. All device types show a similar trend for EPF (23, 23, and 26 meV for devices H, D, and E, respectively). This is in good agreement with the values of the exponential tail states characterized by the Urbach energy of the CZTSSe absorber in each device (Figure S4). However, there is a considerable difference in the BGF values, with γ_BGF_ as high as 188 meV in device D compared to 146 and 125 meV for devices H and E, respectively. This increase can potentially be associated with a higher degree of In diffusion following two successive heat treatments of the buffer layers during the fabrication of this device structure (see the Methods section). An in-depth study of the material characteristics of the buffer and CZTSSe absorber layers in each device was conducted to elucidate the cause of the differences in performance observed during electrical characterization of the three device types.

## Discussion

### XRD Analysis

Microstructural analysis was performed by GIXRD on SLG-Mo–CZTSSe, SLG-Mo–CZTSSe–In_2_S_3_, and SLG-Mo–CZTSSe–CdS film stacks. In the GIXRD patterns, depth profile information was obtained by performing detector scans with fixed incidence angles of 0.5, 0.8, 1.0, and 1.5°. By varying the incidence angle, the penetration depth of X-rays is varied accordingly and can be calculated based on α_*i*_ and material properties (see the Supporting Information). Penetration depths for α_*i*_ = 0.5, 0.8, 1.0, and 1.5° were calculated according to the method detailed in the Supporting Information and determined to be ca. 140, 230, 280, and 410 nm, respectively.

**Figure 4:**
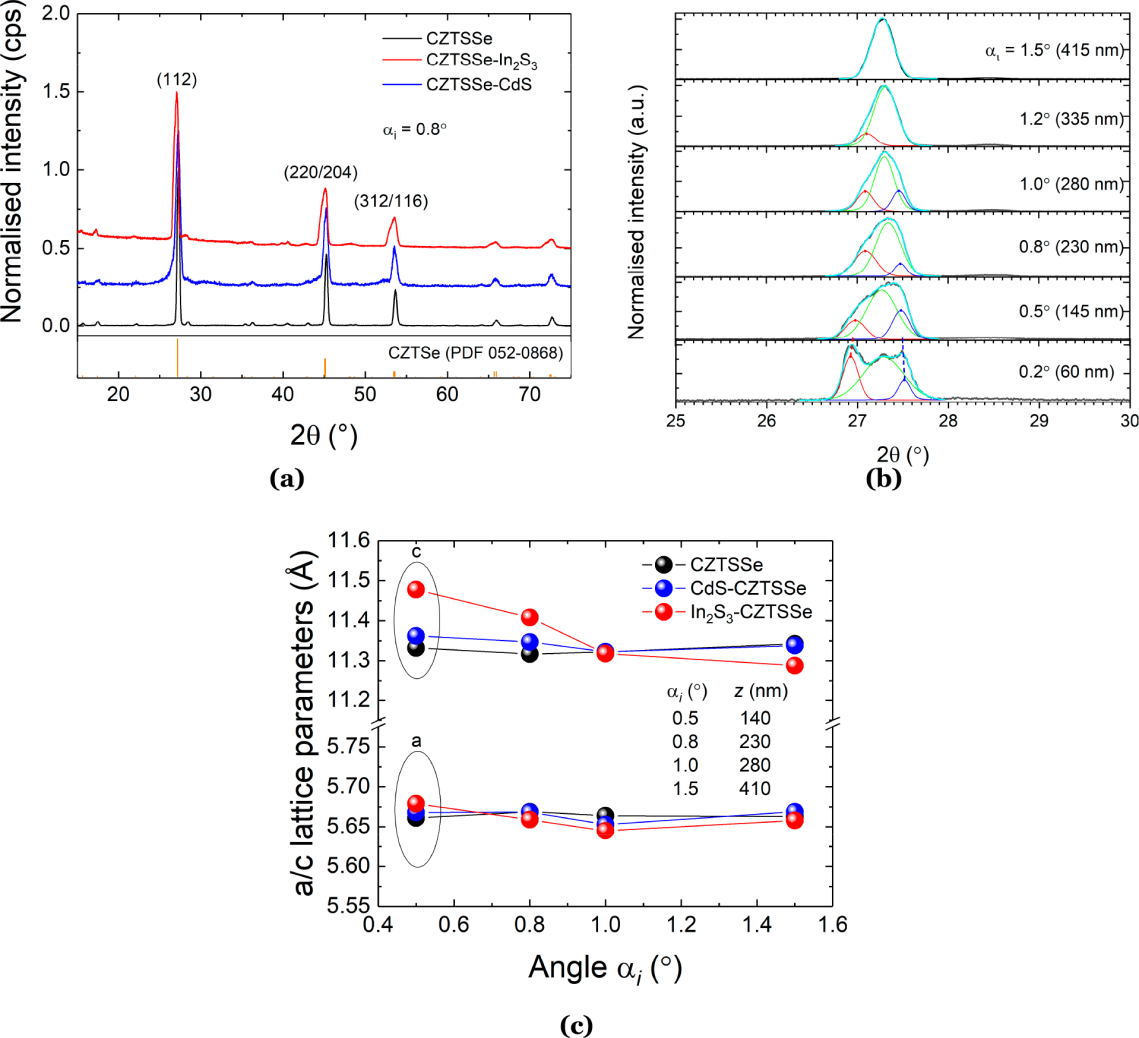
(a) GIXRD patterns of bare CZTSSe, CZTSSe–In_2_S_3_, and CZTSSe–CdS films on Mo-coated SLG at an incidence angle α_*i*_ of 0.5° with peaks referenced to CZTSe (PDF 058-0868). (b) Normalized GIXRD patterns of the (112) peak for the SLG-Mo–CZTSSe–In_2_S_3_ film stack at a series of incidence angles. The corresponding X-ray penetration depths at different incident angles given in parentheses are calculated based on the attenuation law. (c) *a*/*c* lattice parameters of the CZTSSe absorbers in the respective film stacks for the range of α_*i*_ values between 0.2 and 1.5°.

Figure 4a shows the representative diffractograms of film stacks obtained at an angle of 0.5°. All GIXRD spectra reveal distinct peaks of the (112), (220), and (312) planes, which can be assigned to kesterite CZTSe (PDF 052-0868). The peaks for the SLG-Mo–CZTSSe–In_2_S_3_ film stack are broad compared to those of the other film stacks. The (112) peaks of the SLG-Mo–CZTSSe–In_2_S_3_ film stack at a series of incidence angles are therefore normalized and exhibit shoulder peaks, as shown in Figure 4b. When the incidence angle is set at 0.2°, the X-ray penetration depth is calculated to be only ca. 60 nm. This is very close to the actual thickness of the top In_2_S_3_ layer (∼50 nm, as shown in Figure 7b,c) in the film stack. The diffraction pattern therefore provides critical information on the crystal structure of materials at the interface between the CZTSSe absorber and In_2_S_3_ buffer. The diffraction pattern becomes narrow and sharp as the incidence angle increases at the detection region far away from the absorber/buffer interface. Multipeak fits are applied to identify overlapping diffraction peaks of coexistent phases in the broad peak around 27–28°. Apart from the middle main peak belonging to the CZTSSe (112) reflections, there are two shoulder peaks when the incidence angle is shallow. The vertical blue dashed line at a diffraction angle of 27.5° marks the constant position of the β-In_2_S_3_ (311)^42,43^ diffraction peak at various penetration depths. This indicates that the crystal structure of the In_2_S_3_ buffer layer is stable and uniform across the thickness of the thin film. When the incidence angle increases to 1.2° or higher, it is hard to observe the In_2_S_3_ peak anymore because the X-ray detection region is far below the top In_2_S_3_ thin film and minimizes the uncertainty from the surface roughness. In addition to the middle CZTSSe (112) reflections, a shoulder peak at a lower diffraction angle indicates increased interplanar lattice separation, likely due to the incorporation of larger In atoms into the CZTSSe crystal lattice at the absorber/buffer interface. The red dashed line indicates continuous evolution of the shoulder peak as the X-ray penetration depth increases away from the interface and less elemental incorporation into the CZTSSe absorber. When the incidence angle increases to 1.5°, no shoulder peak can be observed, indicating that elemental diffusion is constrained in the narrow interface region only. A shoulder on the main (112) CZTSSe peak in the SLG-Mo-CZTSSe-CdS film is also evident at lower incidence angles, which corresponds to the (111) peak in CdS (PDF 01-075-0581; Figure S5). The CdS peak also disappears as the X-ray penetration depth increases. Complementary techniques SIMS and EDX were performed to validate this hypothesis.

In addition to the peak broadening, the shifting of the XRD peaks to lower values also implies an increase in the lattice parameter values, which can be further explained by the incorporation of an atom with a larger ionic radius into the CZTSSe crystal lattice. Because all CZTSSe absorbers were fabricated from the same batch of CZTS nanoparticle inks and subject to the same selenization conditions, the shift to lower XRD diffraction angles in the stack with a In_2_S_3_/CdS buffer infers elemental diffusion from the buffer to the CZTSSe layer. This phenomenon is well documented in kesterite films, where an increasing [Se]/([S] + [Se]) ratio shifts the CZTS 2θ values from 28.44 to 27.16° in CZTSe, given the larger atomic radius of Se (atomic radius ∼ 1.98 Å) in comparison to s (atomic radius ∼ 1.84 Å).^13,44−47^

As presented in Figure 4c, the CZTSSe lattice parameters (*a* = *b* ≠ *c*) for each device type were calculated using the relationships between the Bragg angle 2θ, interplanar spacing *d*, and lattice parameter *a*, where 1/*d*^2^ = (*h*^2^ + *k*^2^)/*a*^2^ and (*hkl*) is the Miller index of the diffracted plane. There is evidence of changes up to a depth of ∼230 nm in the lattice structure of the CZTSSe absorber in the film stack with an In_2_S_3_ buffer layer. The lattice parameters were increased from *a* = 5.66 to 5.68 Å and from *c* = 11.33 to 11.48 Å from the bare CZTSSe absorber to the In_2_S_3_-buffered absorber. In theory, substitution of the larger In^3+^ ion (ionic radius = 0.80 Å) with the smaller Sn^4+^ ion (ionic radius = 0.69 Å) will cause a systematic diffraction angle shift to lower values.^48−50^ Therefore, it is speculated that In diffuses into the CZTSSe region near the In_2_S_3_/CZTSSe absorber interface following buffer deposition and may induce morphological changes in the CZTSSe absorber near the interface. In the case of the CZTSSe film with a CdS buffer, there is a small increase in the *a*/*c* parameters of the CZTSSe absorber in the near-interface region. This observation again infers the incorporation of an element with a larger ionic radius into the CZTSSe crystal lattice near the buffer/absorber interface. A previous study found that Cd_Cu_ and/or Cd_Zn_ antisite defects formed up to several hundred nanometers from the heterointerface within the CZTS absorber following a postdeposition heat treatment (PDHT) of the CdS/CZTS film.^2^

### SIMS Analysis

**Figure 5:**
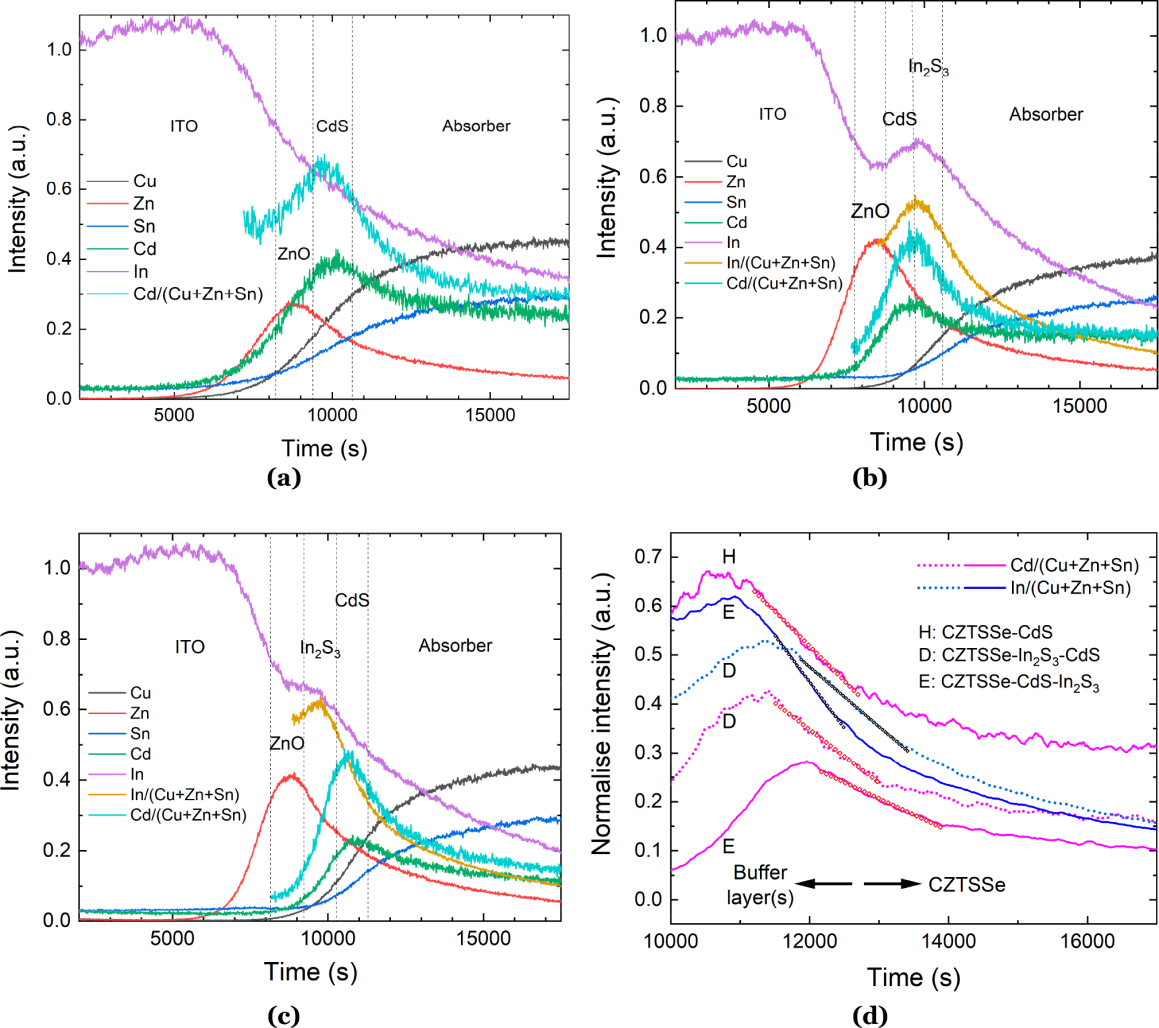
SIMS depth profiles of three solar cells with (a) H: CZTSSe–CdS and (b) D: CZTSSe–In_2_S_3_–CdS, and (c) E: CZTSSe–CdS–In_2_S_3_ stacking at the p–n junction, respectively. Both S and Se signals are excluded, and only metallic component elements are included in the figures to reduce the complexity. All data are normalized using the In signal because both samples have ITO layers deposited in the same sample batch. In/metal and Cd/metal ratio curves are used to help to identify different layers, especially the interfaces in solar cells. (d) Slopes of the Cd/metal and In/metal curves in both samples D and E to understand the elemental diffusion at the buffer/absorber interfaces.

Figure 5 shows the SIMS depth profiles near the absorber/buffer interface for all device structures. In Figure 5b, we can clearly distinguish In_2_S_3_ and CdS layers with the aid of the Zn signal and In/metal and Cd/metal peaks.

As shown in Figure 5b, the buffer/absorber interface is determined using the peak of the Cd/metal curve. A more detailed discussion of the determination of the buffer/absorber interface is presented in Figure S6. The dashed vertical lines therefore divide the profile into a series of composition zones from the top surface of the solar cell top surface: indium-doped tin oxide (ITO)/ZnO/In_2_S_3_/CdS/CZTSSe absorber. In Figure 5c, however, the Cd and In signals overlap with each other at the p–n junction region. There is only a small shift between the peaks of the Cd and In signals. This indicates that some elemental diffusion might exist at the buffer/absorber interface region. Therefore, the slopes of the In/metal and Cd/metal curves near the buffer/absorber interface are studied in Figure 5d to further understand the elemental diffusion at the p–n junction interface. It can be seen that the slope of the In signal in device type E is sharper (absolute slope = 1.85 × 10^–4^) than that of device D (absolute slope = 1.32 × 10^–4^). This means that the In signal decays slower in sample D and indicates more In diffused into the bottom CZTSSe absorber.

### Electron Microscopy and Composition

**Figure 6:**
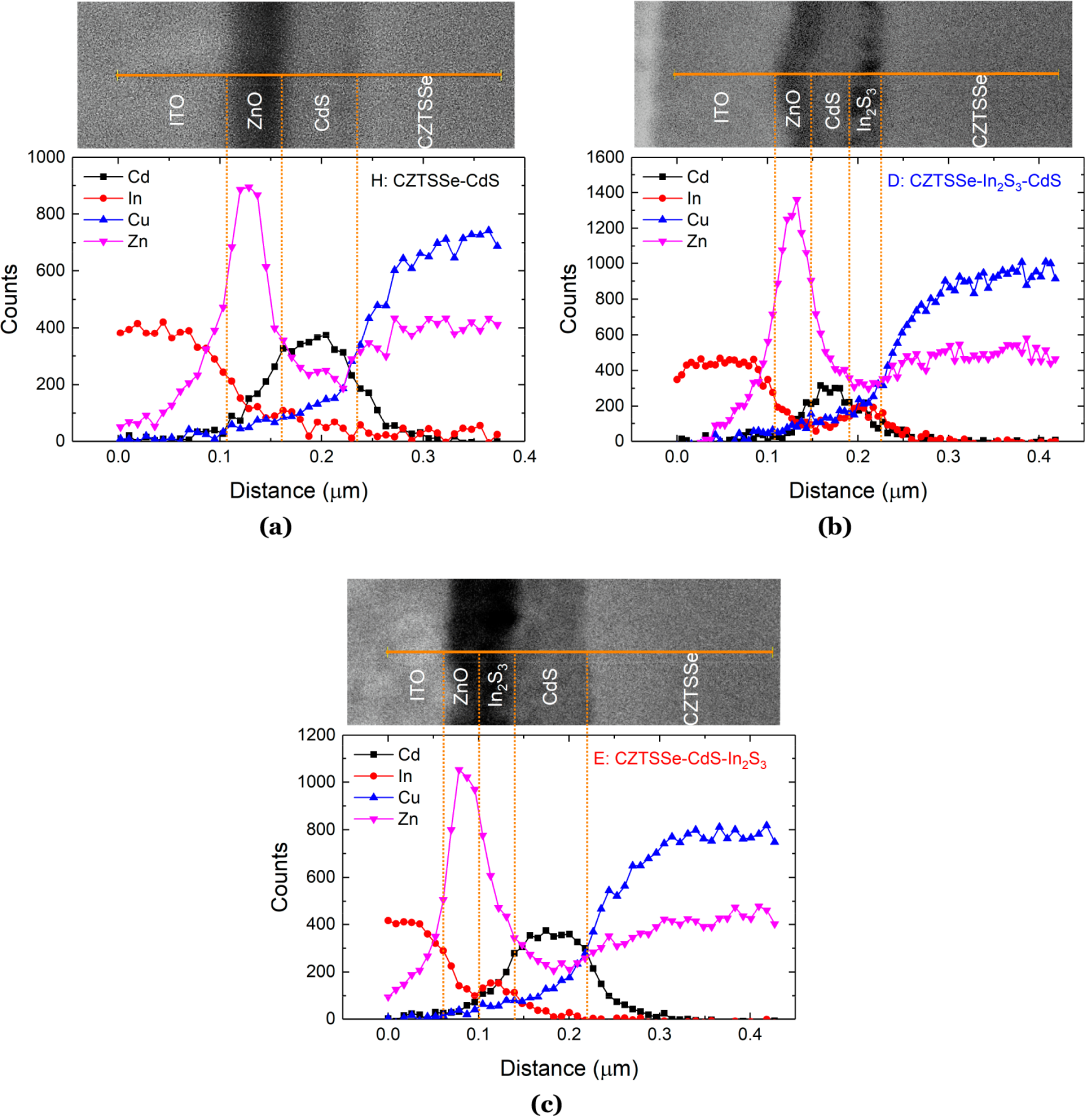
EDX line scans across the buffer/absorber interfaces of devices (a) H: CZTSSe– CdS, (b) D: CZTSSe–In_2_S_3_–CdS, and (c) E: CZTSSe-CdS-In_2_S_3_, together with the BSE images of the corresponding FIB device cross sections.

EDX mapping and line scans were performed to identify the chemical composition and distribution of the different layers in the region around the buffer/absorber interface (Figure 6). Included in the figures are the corresponding SEM cross sections of the relevant devices obtained by backscattered electron (BSE) imaging. The BSE image contrast depends on the average atomic weight of the elements in the constituent layers; i.e., materials with a higher average atomic weight appear lighter in the BSE images. As such, the individual ITO, ZnO, In_2_S_3_, CdS, and CZTSSe layers are clearly distinguishable in all device-type cross sections, and EDX elemental profile data are presented corresponding to the orange line through the cross sections. There is evidence of elemental interdiffusion across the heterojunction of the buffer(s)/CZTSSe films in all device types. Specifically, there is significant diffusion of the metallic element in the buffer (In or Cd) into the absorber when the buffer adjoins the CZTSSe layer. Diffusion of the metallic elements from the buffer to the absorber is promoted by the application of a postdeposition heat treatment following chemical bath deposition (CBD).^2,19^ Researchers at the University of New South Wales found that, following a heterojunction heat treatment (300 °C for 10 min in N_2_ atmosphere) of CZTS directly after CBD of CdS, the subsequent CZTS device efficiency improved from ∼8% to 11%. The improvement was directly attributed to Cd diffusion into the CZTS absorber up to a depth of ca. 200 nm, forming Cd_Cu_ or Cd_Zn_ antisite defects and Zn diffusion into the CdS layer.

**Figure 7:**
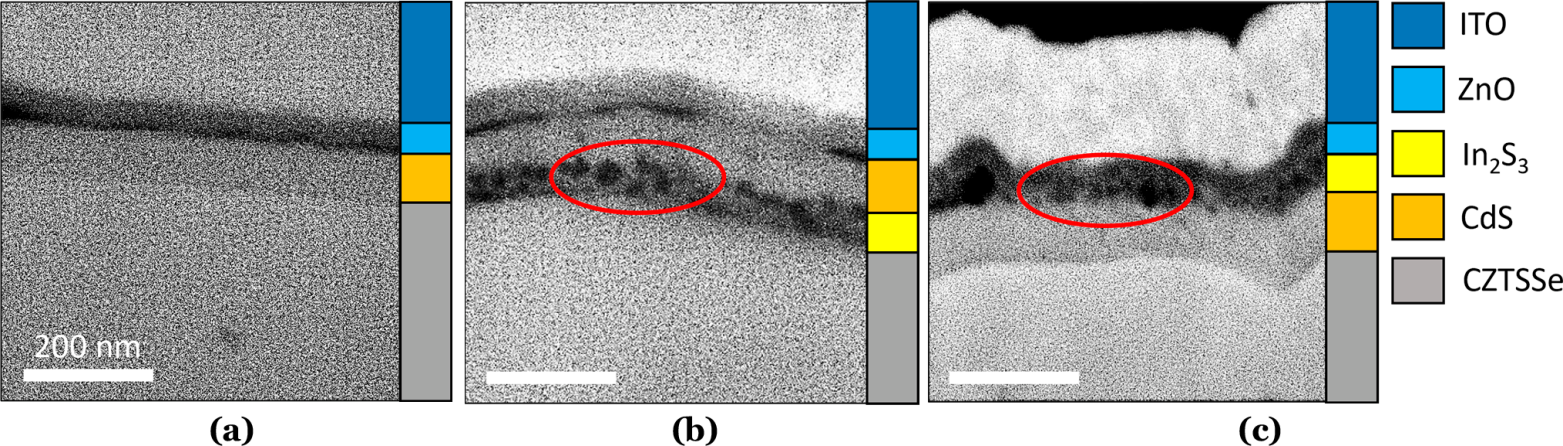
BSE images of device cross sections for (a) H: CZTSSe–CdS, (b) D: CZTSSe–In_2_S_3_–CdS, and (c) E: CZTSSe–CdS–In_2_S_3_ solar cells. Heavier elements/films appear as lighter areas in contrast to the images, which enables identification of the individual layers within the structure. The highlighted areas in parts b and c show regions of intermixing of the CdS and In_2_S_3_ layers.

As a result, thin layers of Cu_2_Cd_*x*_Zn_1*–x*_SnS_4_ and Cd_*x*_Zn_1*–x*_S formed around the buffer/absorber interface, improving the energy-band alignment at the junction and reducing interface recombination.^2^Figure 7 shows BSE images of the top window–buffer–absorber layers of the respective CZTSSe solar cells. In both devices with a dual buffer, there is evidence of intermixing of the layers at the CdS/In_2_S_3_ interface. This intermixing of the buffer layers may arise from the successive heat treatments (200 °C in air for 10 min), following deposition of the individual buffers.

### Electron-Beam-Induced Current (EBIC)

**Figure 8:**
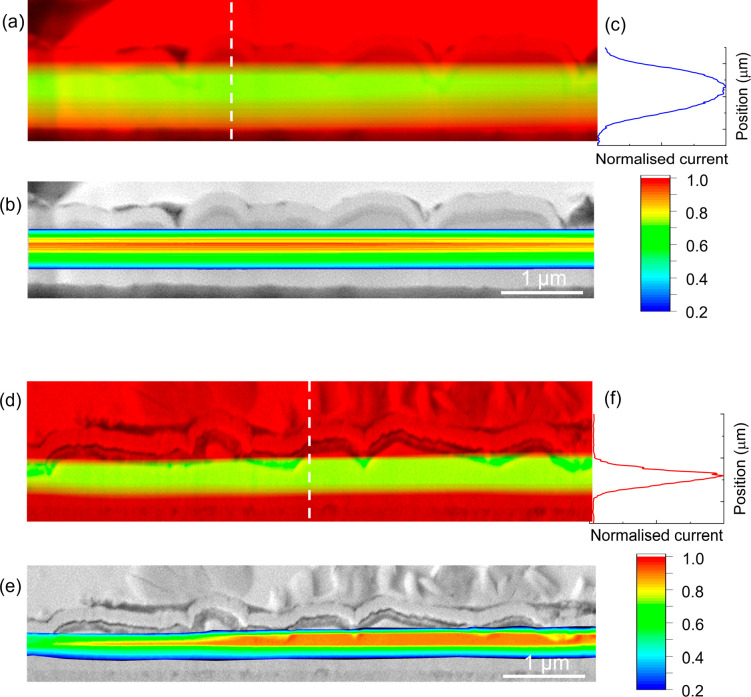
(a and d) SEM cross-sectional images of devices D: CZTSSe–In_2_S_3_–CdS and E: CZTSSe–CdS–In_2_S_3_ (red) with a superimposed EBIC signal (green). (b and e) Same SEM cross-sectional images with color maps of the EBIC signals to highlight variations in the signal intensity (signal intensities below 0.2 are not shown to aid visualization). (c and f) Normalized extracted EBIC profiles based on the respective line scans indicated by dashed lines in parts a and d.

The charge-carrier collection behavior in the devices with a dual buffer was investigated by means of EBIC measurements on flat cross sections obtained by focused-ion-beam (FIB) milling (Figure S7). EBIC measurements visualize the depth-dependent collection of photogenerated carriers in the semiconductor materials in the CZTSSe solar cells. The SEM images (red) are overlaid with the normalized EBIC signal (green), as shown in parts a (device D) and d (device E) of Figure 8. The brightest areas in the EBIC signal show regions of higher current collection over the scanned area of the device cross section and are a measure of the electric field that separates the generated electron–hole (e–h) pairs. The peak of the electric field appears to be broader and generated deeper within the CZTSSe absorber bulk in device D compared to device E. Color maps of the EBIC signals were created to detail the variation in the signal intensity across the device cross sections (Figure 8b,e). For better visualization, EBIC signals below 0.2 are not shown. In the case of device E, a large area of relatively high EBIC signals (red region of the color map) is observed within the CZTSSe absorber close to the CdS/CZTSSe interface, compared to a narrower region of high signal intensity observed in device D (Figure 8b,e). Because the SCR width in device E is ca. 80 nm (determined from *C*–*V* measurements), the e–h pairs generated deeper inside the absorber bulk have to diffuse greater distances in order to be effectively separated by the electric field associated with the p–n junction. The higher EBIC signal extending over a larger area in the CZTSSe absorber of device E suggests that the minority carriers (electrons) have a greater minority carrier diffusion length (*L*_D_) than counterpart device D. Approximate *L*_D_ values were determined from the absorption coefficient data for the CZTSSe absorbers and the EQE spectra for the respective devices (see the Supporting Information and Figure S8). Subsequent *L*_D_ values of ca. 120 and 300 nm for devices D and E were obtained, respectively, which are in good agreement with the observed EBIC signals generated in the device cross sections. Similar results have also been reported for kesterite solar cells.^51−53^ EBIC line profiles perpendicular to the p–n junction were extracted at positions indicated by the dashed white lines on the SEM images and the corresponding normalized integrated EBIC signal (*I*_EBIC,norm_) displayed in Figure 8c,f. The EBIC signal in device D presents as a broad signal expanding almost the full width of the CZTSSe absorber, with its peak deep within the bulk.

**Figure 9:**
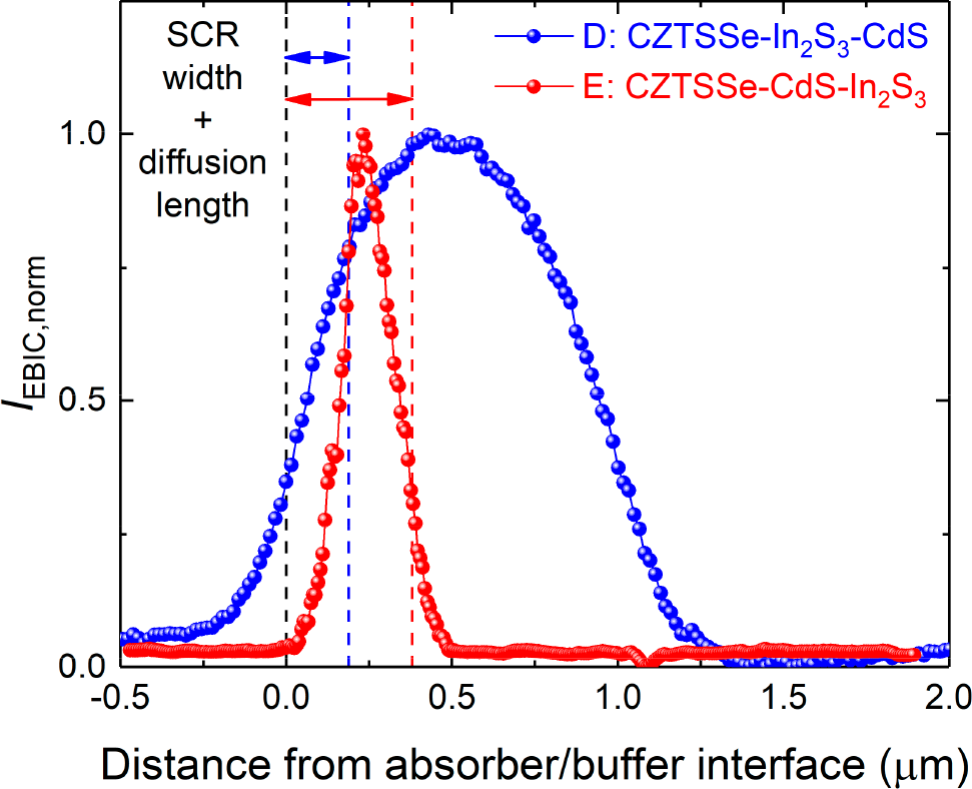
Quantitative EBIC current data as a function of the beam position relative to the devices D: CZTSSe–In_2_S_3_–CdS and E: CZTSSe–CdS–In_2_S_3_ extracted from the respective line scans in Figure 8a,d. The dashed red and blue lines represent the sum of the SCR width and minority carrier diffusion length for each device determined from *C*–*V* and EQE measurements, respectively.

In contrast, device E has an EBIC line profile with a narrow peak located closer to the buffer/absorber interface. For comparison, EBIC line profiles were plotted in relation to their position from the buffer/CZTSSe interface for both device types (Figure 9). The dashed lines in the figure represent the optimal carrier collection lengths *w*_SCR_ + *L*_d_ within which the generated e–h pairs from the impinging electron beam can be separated and extracted from the devices. The peak of the electric field in device E lies within this region, leading to improved charge separation and carrier transport and, consequently, a higher *J*_SC_.

The opposite effect is true for device D, where peak e–h generation is deeper with the absorber bulk and is susceptible to higher carrier recombination. This could account, in part, for the lower *J*_SC_ in these devices compared with E devices. Additionally, the raw EBIC signal observed in D was lower than that in E considering the identical measurement conditions for both types of devices (Figure S9).

## Conclusion

In summary, CZTSSe solar cells with a combination of CdS and In_2_S_3_ buffer layers were compared with standard devices with a single CdS layer. Devices with the structure CZTSSe–CdS–In_2_S_3_ generally performed better than standard devices, with the best device achieving a maximum conversion efficiency of 7.75% (an increase of over 10% compared to the best standard device). Conversely, devices with a CZTSSe–In_2_S_3_–CdS structure typically demonstrated lower *V*_OC_ and, as a consequence, had lower overall efficiency compared to standard and CZTSSe–CdS–In_2_S_3_ devices. CZTSSe–CdS–In_2_S_3_ devices also showed an appreciable reduction in reverse saturation currents in the SCRs and QNRs of the CZTSSe absorbers. Compositional analysis of the absorber/buffer interfacial region of the CZTSSe solar cells identified significant Cd diffusion from CdS into the CZTSSe absorber in the CZTSSe–CdS–In_2_S_3_ device and is correlated to the double postdeposition heat treatment of the CdS layer during dual buffer deposition. Cd diffusion has been shown to be beneficial to the device performance by forming a thin layer of Cu_2_Cd_*x*_Zn_1–*x*_(S,Se)_4_ on the absorber face, which promotes a more favorable band alignment. Consequently, recombination at the heterojunction region is significantly reduced. The opposite is true for devices with a CZTSSe–In_2_S_3_–CdS structure. In diffusion into the CZTSSe absorber is also observed as a result of the double heat treatment of the In_2_S_3_ layer, which serves to increase the p-type doping of the absorber believed to be caused by the formation of acceptor antisite defects In_Sn_. The width of the SCR is reduced consequently. EBIC measurements revealed that peak e–h generation occurred closer to the p–n (absorber/buffer) junction in the CZTSSe–CdS–In_2_S_3_ device, which facilitates charge extraction due to the larger “effective” diffusion length of minority carriers in this device. Conversely, maximum e–h generation takes place deeper into the bulk of the CZTSSe absorber of the CZTSSe–In_2_S_3_–CdS solar cell, which suffers from a shorter “effective” diffusion length and a higher recombination in the SCR. The device performance is negatively impacted by these factors. This study demonstrates the importance of a quality absorber/buffer interface in achieving efficient solar cells and the positive effects of Cd diffusion on the CZTSSe device performance.

## Methods

### CZTS Nanoparticle Inks

CZTS nanoparticles were fabricated using a hot-injection method where a sulfur–oleylamine (OLA) solution was injected into a hot metallic precursors–OLA solution under air-free conditions. The metallic precursor molar ratios were chosen to be Cu/(Zn + Sn) = 0.79 and Zn/Sn = 1.27, achieved by using 1.34 mmol of Cu(acac)_2_, 0.95 mmol of Zn(acac)_2_, and 0.75 mmol of Sn(acac)_2_Cl_2_ (where acac = acetylacetonate) as the metallic source to guarantee a Cu-poor, Zn-rich composition region for high solar cell efficiencies. After a reaction at 225 °C for 30 min, the as-synthesized nanoparticles were precipitated and washed twice by using isopropyl alcohol and toluene. The collected CZTS nanoparticles were dispersed with the aid of sonication to provide CZTS nanoparticle inks with a concentration of ∼200 mg/mL. More details have been described in our previous works.^7,13^

### Thin-Film Deposition

The resulting nanoparticle inks were deposited on Mo–glass substrates via spin-coating.^24^ Approximately 30 μL of the concentrated ink was applied onto a square (2.5 cm × 2.5 cm) Mo-coated glass substrate at a speed of 1200 rpm for 5 s. The samples were then dried on a hot plate at 150 °C for 30 s and then at 300 °C in air for 30 s (hereafter “soft-baking”) to remove the residual solvents. The thickness of the deposited thin films could be accurately controlled and reproduced by repeated spin-coating and soft-baking procedures. A thickness of ∼1 mm was set for efficient light absorption in all thin films.

### Dual Buffer Structure

CBD was used to deposit the buffer layers of CdS and In_2_S_3_. Specifically, a CdS thin film was fabricated by using cadmium sulfate as the Cd source, thiourea as the S source, and ammonium hydroxide to adjust the pH around 11.9. In terms of In_2_S_3_ deposition, samples were immersed in a solution composed of indium chloride (10 mM), thioactamide (0.1 M), and acetic acid (0.1 M) at 70 °C to deposit an In_2_S_3_ coating on the CZTSSe absorber, with details given in elsewhere.^17^ Typical thicknesses of the CdS and In_2_S_3_ layers are ∼70 and ∼50 nm, respectively. For the dual buffer device, a bottom buffer layer was deposited first, followed by a top buffer layer to provide a CdS–In_2_S_3_ or In_2_S_3_–CdS dual buffer structure. After each buffer layer deposition, the samples were removed from the bath, rinsed with deionized water, dried under a nitrogen stream, and then annealed at 200 °C for different times, i.e., CdS for 10 min and In_2_S_3_ for 2 min and 10 min in air.

### PV Device Fabrication

A solar cell device was completed by the addition of the transparent oxide layers, including *i*-ZnO (∼35 nm) and ITO (∼200 nm) layers via magnetron sputtering. Ni (∼50 nm) and Al (∼1 μm) layers were then deposited through a shadow mask by an electron beam to form the front contact grids. Devices were electrically isolated by using mechanical scribing to define a device area of 0.16 mm^2^.

### Solar Cell Characterization

Electrical characterization was performed using a Keithley 2400 sourcemeter and Abet Technologies Sun 2000 solar simulator with an air mass 1.5 spectrum set at 100 mW/cm^2^. EQE measurements were performed using a Betham Instruments PVE300 spectral response system with a W light source (calibrated using a Si-InGaAs reference cell). *C*–*V* measurements were performed using an Agilent E4980a LCR meter at a frequency of 100 kHz with bias voltages from −0.8 to +0.8 V. Elemental depth profiling was performed by SIMS using a primary Ar^+^ beam of 4 keV, a crater area of 500 × 500 μm^2^, and a gating of 10%. For GIXRD measurements, a Siemens D-5000 diffractometer using a Cu Kα radiation source (λ = 0.154 nm) was used at a beam voltage of 40 kV and a beam current of 50 mA in the parallel beam setup. In this work, a Tescan Mira 3 field-emission-gun scanning electron microscope was used for SEM imaging, together with an Oxford Instruments X-Max X-ray spectrometer fitted with a 20 mm^2^ detector operating at 10–20 kV for EDX measurements.

### EBIC Measurement

Samples were prepared for EBIC analysis using FIB cross-section milling with a Ga liquid metal ion source FEI Helios Nano Lab 600 dual-beam system. A series of in situ polishing steps were performed to produce a clean surface with minimal beam damage. Simultaneous EBIC analysis and secondary electron imaging were carried out with a Hitachi SU-70 microscope, with EBIC signals being collected through a Matelect ISM6 specimen current amplifier, using a beam voltage of 5 kV and a current of 0.75 nA. The image filter frequency was set to 10 kHz; this eliminated both noise and fine image detail, and so the images in this paper show the average position of the junctions in the PV devices.

## Supporting Information Available

Double-diode fit to a raw *J*–*V* curve of an example CZTSSe H reference device, built-in voltage *V*_bi_ for all CZTSSe device types, CZTSSe absorber band-gap *E*_G_ determination from the EQ spectra for all CZTSSe device types, X-ray penetration depth method and calculation, alignment of the SIMS profiles using the Zn peak for all CZTSSe samples, SEM FIB cross sections for dual buffer CZTSSe devices, minority carrier diffusion length calculation from the EQE spectra for all CZTSSe devices, and raw EBIC line scan data for dual buffer CZTSSe devices (PDF)
(ZIP)
