# Supplementary material for: Enhanced Carrier Collection in Cd/In-Based Dual Buffers in Kesterite Thin-Film Solar Cells from Nanoparticle Inks
Source: ACS Appl Energy Mater. 2023 Oct 27;6(21):10883–96. doi: 10.1021/acsaem.3c01622 (PMC10646902; doi:10.1021/acsaem.3c01622)
Supplement: Supplementary file 1 — ae3c01622_si_001.pdf [file ae3c01622_si_001.pdf]

Supporting Information

Enhanced carrier collection in Cd/In-based dual buffers in kesterite thin film solar cells from nanoparticle inks

Stephen Campbell,† Guillaume Zoppi,† Leon Bowen,‡ Pietro Maiello,† Vincent Barrioz,† Neil S. Beattie,† and Yongtao Qu*,†

†*Dept. of Mathematics, Physics and Electrical Engineering, Northumbria University, Newcastle-upon-Tyne, NE1 8ST, United Kingdom*

‡*Dept. of Physics, Durham University, Durham, DH1 3LE, United Kingdom*

E-mail: y.qu@northumbria.ac.uk

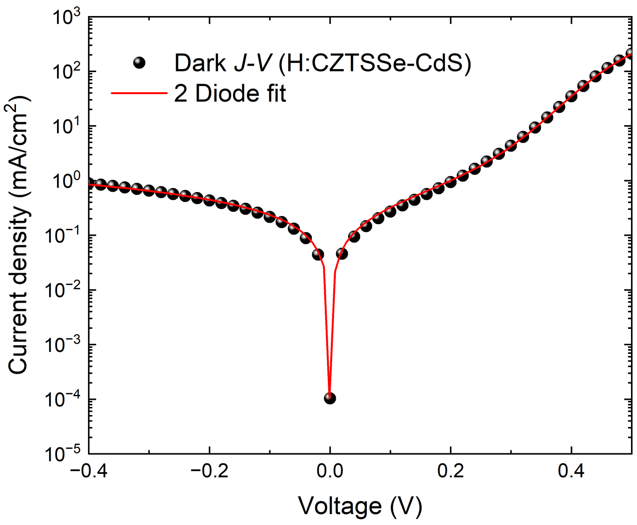

Figure S1: Example of double diode fit according to Eq. (1) of a dark $J-V$ curve for device H:CZTSSe-CdS.

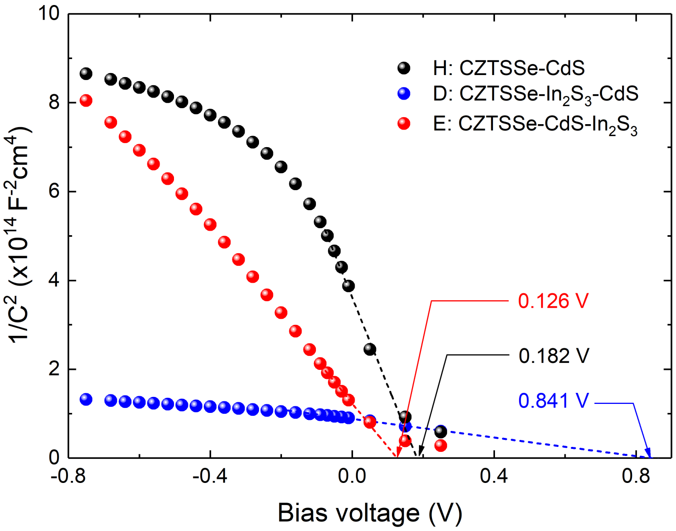

Figure S2: Mott-Schottky analysis of all CZTSSe device structures with built-in voltage (V_{bi}) indicated for respective devices.

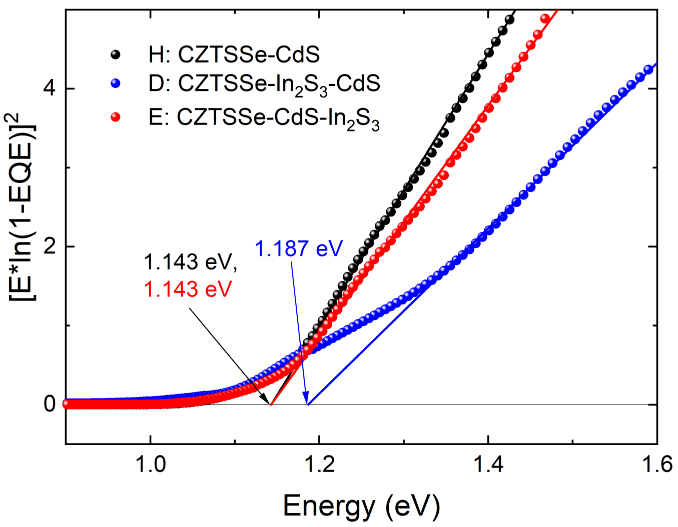

Figure S3: Bandgap (E_G) determination from EQE data for all device structures.

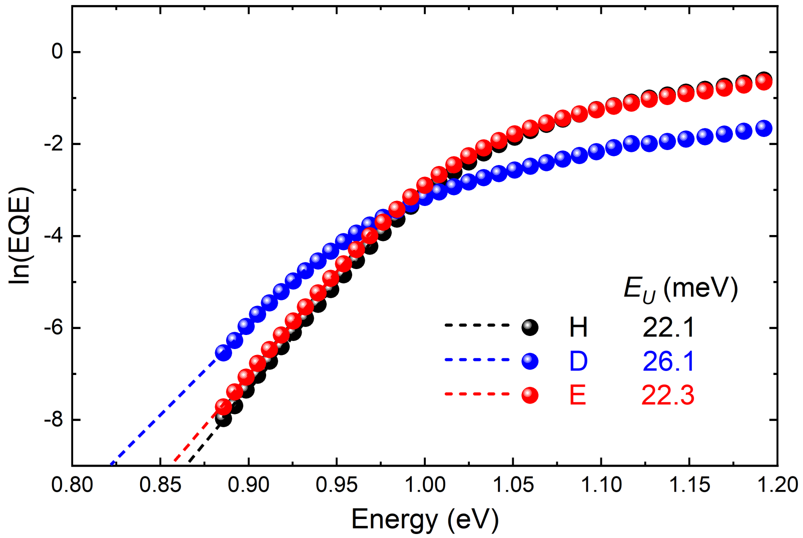

Figure S4: Urbach energy (E_U) evaluation from EQE data for all device structures.

X-ray penetration depth calculation

Based on the attenuation law, the penetration depth z in kesterite materials can be calculated according to the equation:

$$z = \frac{1}{\mu}\left[\frac{1}{sin(\alpha_i)} + \frac{1}{sin(2\theta - \alpha_i)}\right]^{-1} \tag{S1}$$

where α_i is the incidence angle, 2θ is the Bragg angle and μ is the attenuation coefficient. To determine the attenuation coefficient for kesterite compounds, the overall attenuation coefficient of the compound was obtained by addition of the elemental mass attenuation coefficients, $(\mu/\rho)_i$:

$$\mu(CZTSSe) = \rho(CZTSSe) \sum_i \frac{M_i}{M(CZTSSe)} (\mu/\rho)_i \tag{S2}$$

where M_i is the mass fraction of the specific element in the CZTSSe compound, $M(CZTSSe)$ is the total mass of the CZTSSe molecule and $\rho(CZTSSe$ is the density of the CZTSSe compound which is given by:

$$\rho(CZTSSe) = x\rho(CZTS) + (1 - x)\rho(CZTSe) \tag{S3}$$

where x is the [S]/([S]+[Sr]) ratio. Values of 4.57 g/cm^3 and 5.69 g/cm^3 were used for the density of CZTS and CZTSe, respectively[1] and elemental mass attenuation coefficients, $(\mu/\rho)_i$ for Cu, Zn, Sn, S and Se taken from National Institute for Standards and Technology (NIST)[2]

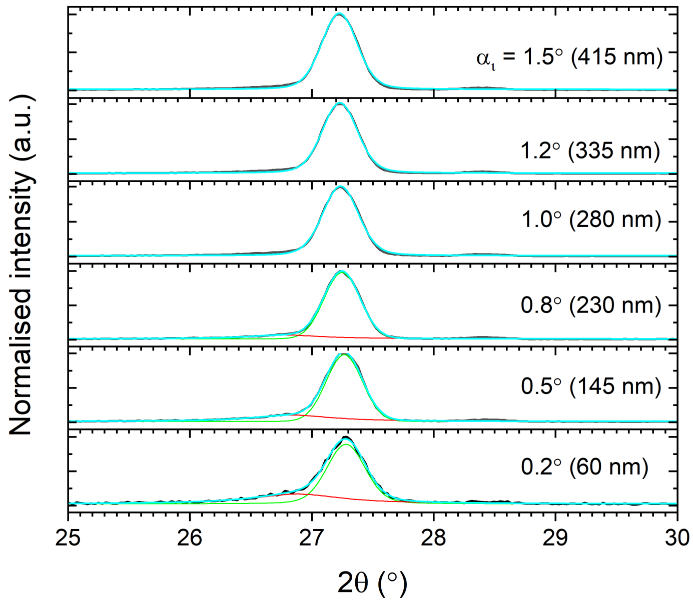

Figure S5: Normalised GIXRD patterns of SLG-Mo-CZTSSe-CdS film stack at a series of incidence angles α_i between 0.2° and 1.5°. The corresponding X-ray penetration depth at different incident angles are calculated based on the attenuation law. The shoulder peak around $2\theta = 26.88°$ can be correlated to the (111) peak in CdS PDF reference 01-075-0581.

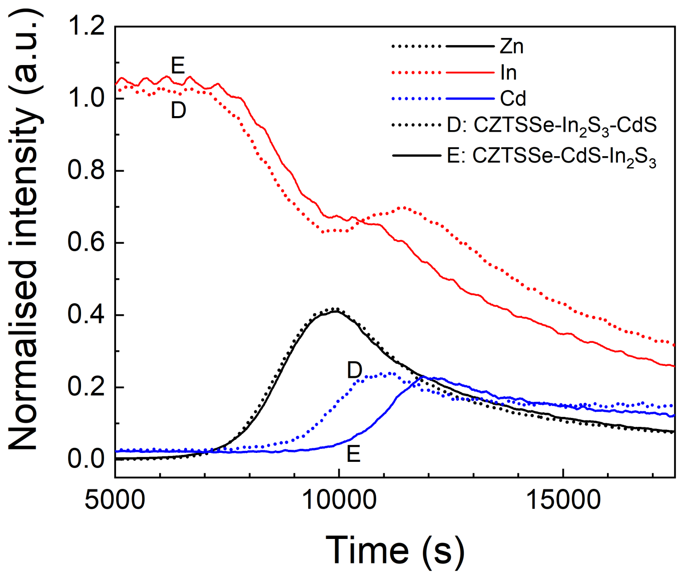

Figure S6: SIMS profiles of devices D:CZTSSe-In_2S_3-CdS and E:CZTSSe-CdS-In_2S_3 showing Zn, Cd and In signals. The elemental spectra were shifted to align the Zn signals of both devices to enable clearer identification of the structure interfaces.

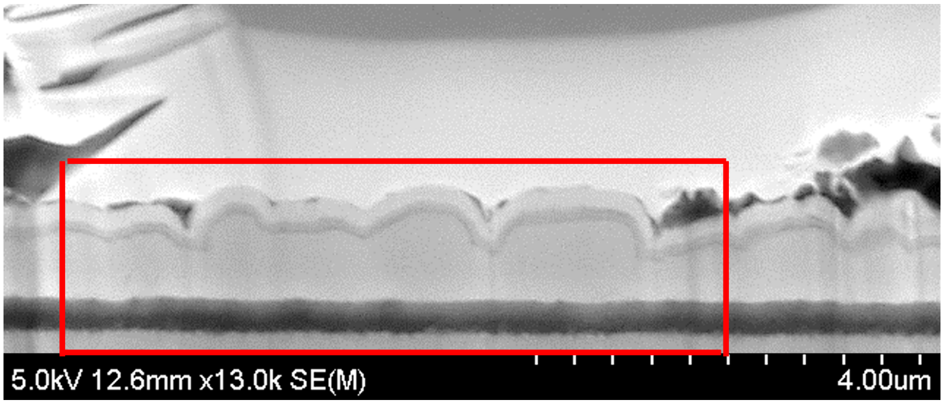

(a)

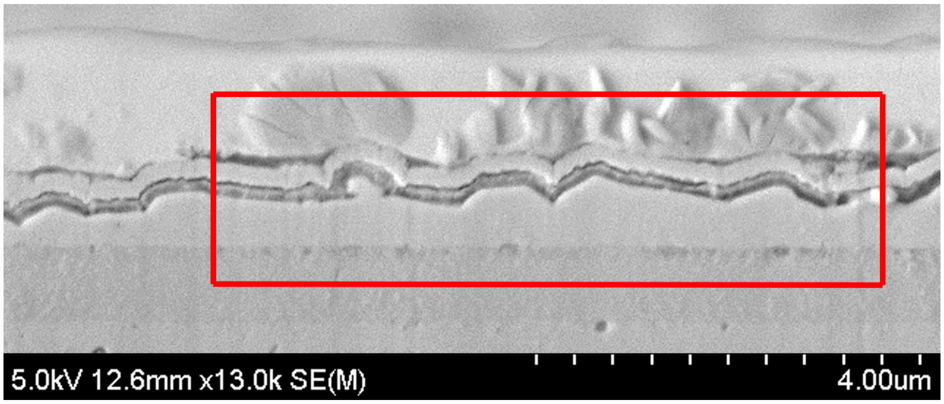

(b)

Figure S7: SEM cross-sectional images (obtained by focussed ion beam milling) of devices (a) D:CZTSSe-In_2S_3-CdS and (b) E:CZTSSe-CdS-In_2S_3. The red box shows the device cross-sectional area used in Figure 8.

Minority carrier diffusion length determination

The minority carrier diffusion length (L_D) for the CZTSSe absorbers was calculated from external quantum efficiency (EQE) measurements in conjunction with optical absorption coefficient (α) measurements (determined from transmittance/reflectance of the CZTSSe films) using a method by Courel *et al.*[3] It was shown that 1/EQE is a linear function of $1/(\alpha)$:

$$\frac{1}{EQE(\lambda)} = \frac{1}{(1 - R(\lambda))}\left[1 + \frac{(1/\alpha(\lambda))}{L_d}\right] \tag{S4}$$

where $R(\lambda)$ is the reflectance of the photovoltaic device with an intercept on the $1/\alpha$ axis

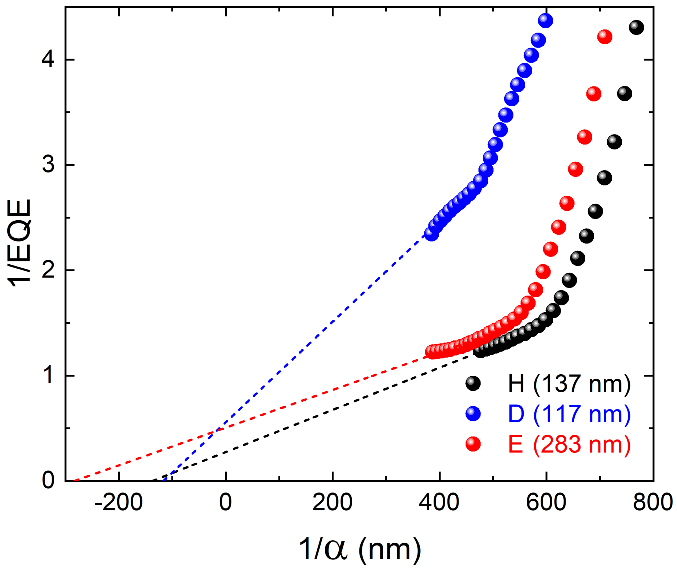

Figure S8: Minority carrier diffusion length determination obtained from absorption coefficient and EQE data for all device structures according to Equation S1.

equal to L_D. Diffusion lengths of 137, 117 and 297 nm were determined for devices H: CZTSSe-CdS, D: CZTSSe-In_2S_3-CdS and E: CZTSSe-CdS-In_2S_3, respectively.

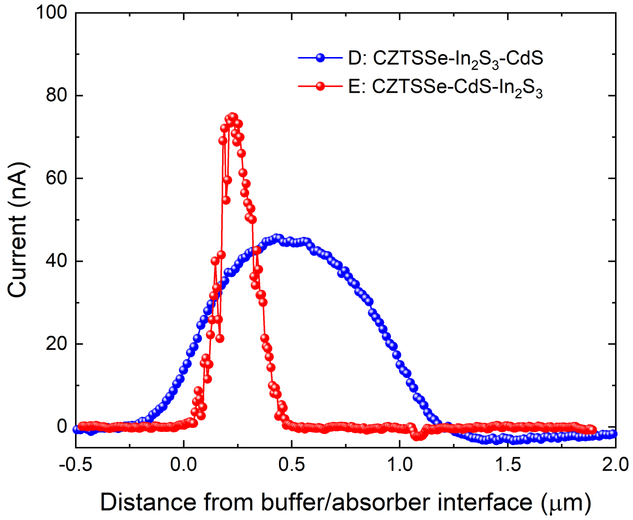

Figure S9: Raw EBIC line scan data for devices D:CZTSSe-In_2S_3-CdS and E:CZTSSe-CdS-In_2S_3 under the same measurement conditions.

References

(1) Guen, L.; Glaunsinger, W. Electrical, magnetic, and EPR studies of the quaternary chalcogenides $Cu_2A_{II}B_{IV}X_4$ prepared by iodine transport. *Journal of Solid State Chemistry* **1980**, *35*, 10–21.

(2) Chantler, C. T.; Zucker, D. S.; Kotochikova, S. A.; Kishore, A. R.; Chang, J.; Dragoset, R. A.; Olsen, K. X-ray form factor, attenuation, and scattering tables. 2022; https://dx.doi.org/10.18434/T4HS32.

(3) Courel, M.; Valencia-Resendiz, E.; Pulgarín-Agudelo, F.; Vigil-Galán, O. Determination of minority carrier diffusion length of sprayed-Cu_2ZnSnS_4 thin films. *Solid-State Electronics* **2016**, *118*, 1–3.
