# Supplementary figures and images for: Enhanced Carrier Collection in Cd/In-Based Dual Buffers in Kesterite Thin-Film Solar Cells from Nanoparticle Inks
Source: ACS Appl Energy Mater. 2023 Oct 27;6(21):10883–96. doi: 10.1021/acsaem.3c01622 (PMC10646902; doi:10.1021/acsaem.3c01622)

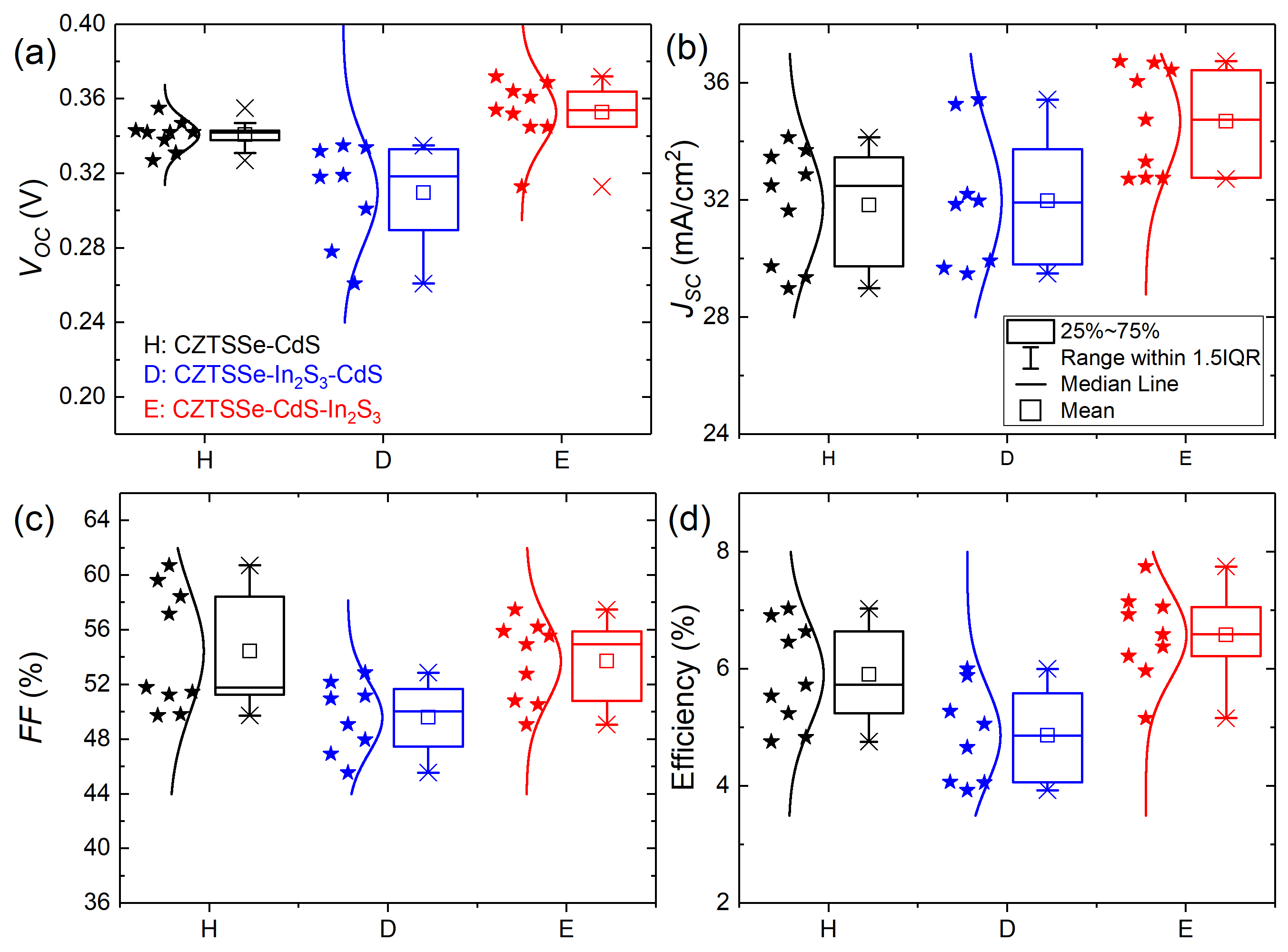

Supplement: Supplementary file 2 — ae3c01622_si_002.zip [file ae3c01622_si_002.zip › Box_plots_JV_curves_with_labels.png]

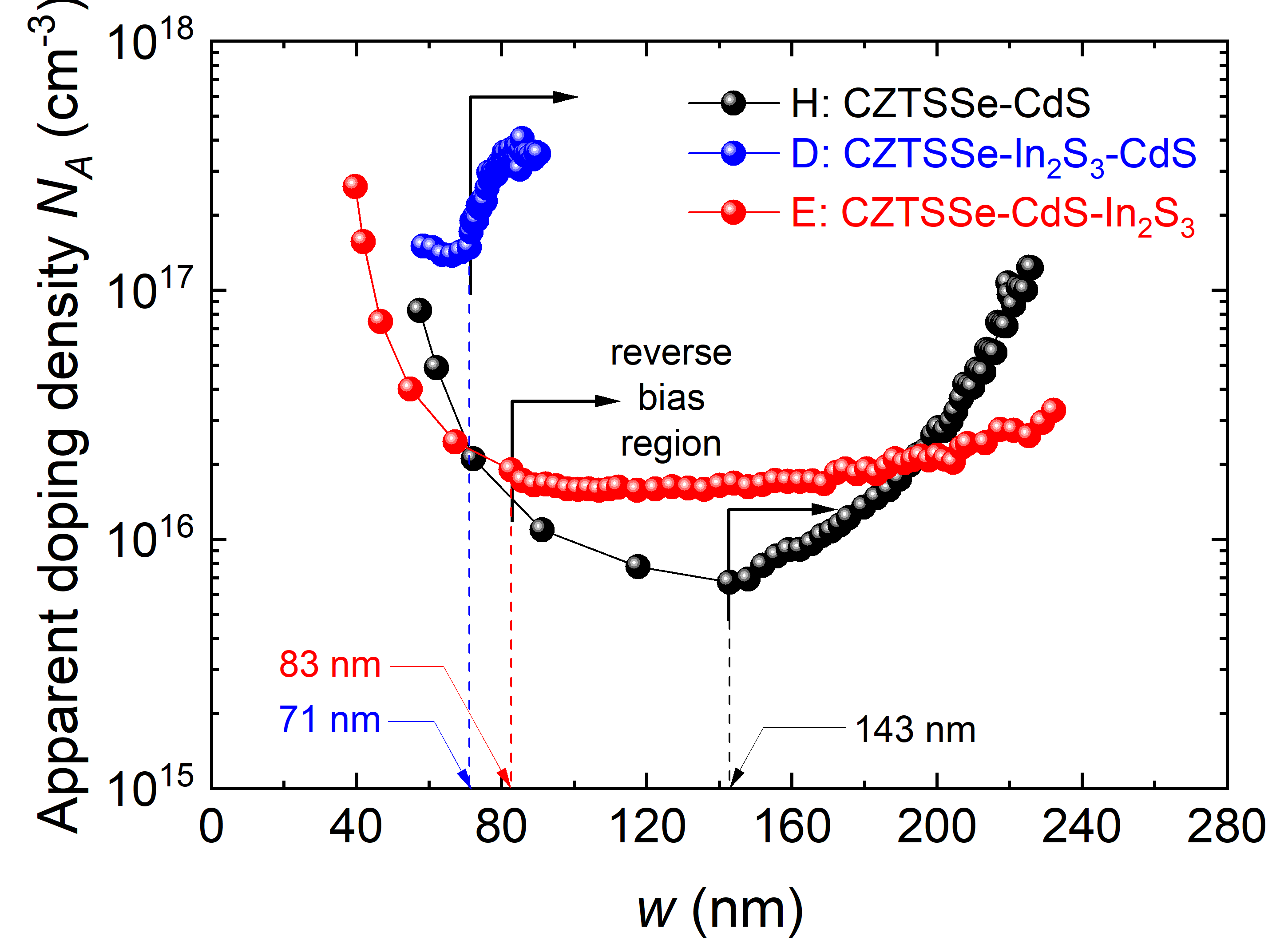

Supplement: Supplementary file 2 — ae3c01622_si_002.zip [file ae3c01622_si_002.zip › CV_dual_buffers_CZTSSe_MRS.png]

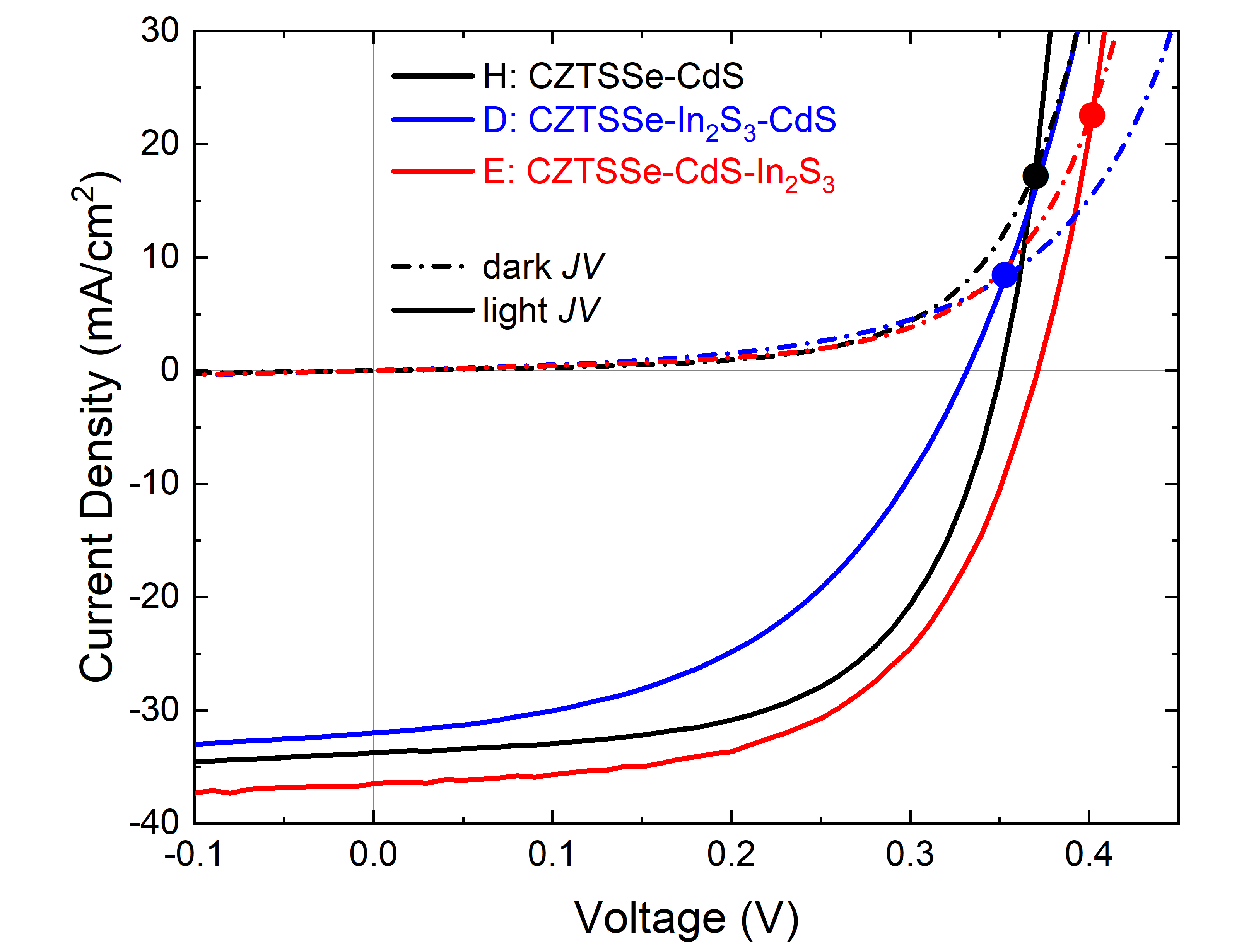

Supplement: Supplementary file 2 — ae3c01622_si_002.zip [file ae3c01622_si_002.zip › JV_crossover_dual_buffers.png]

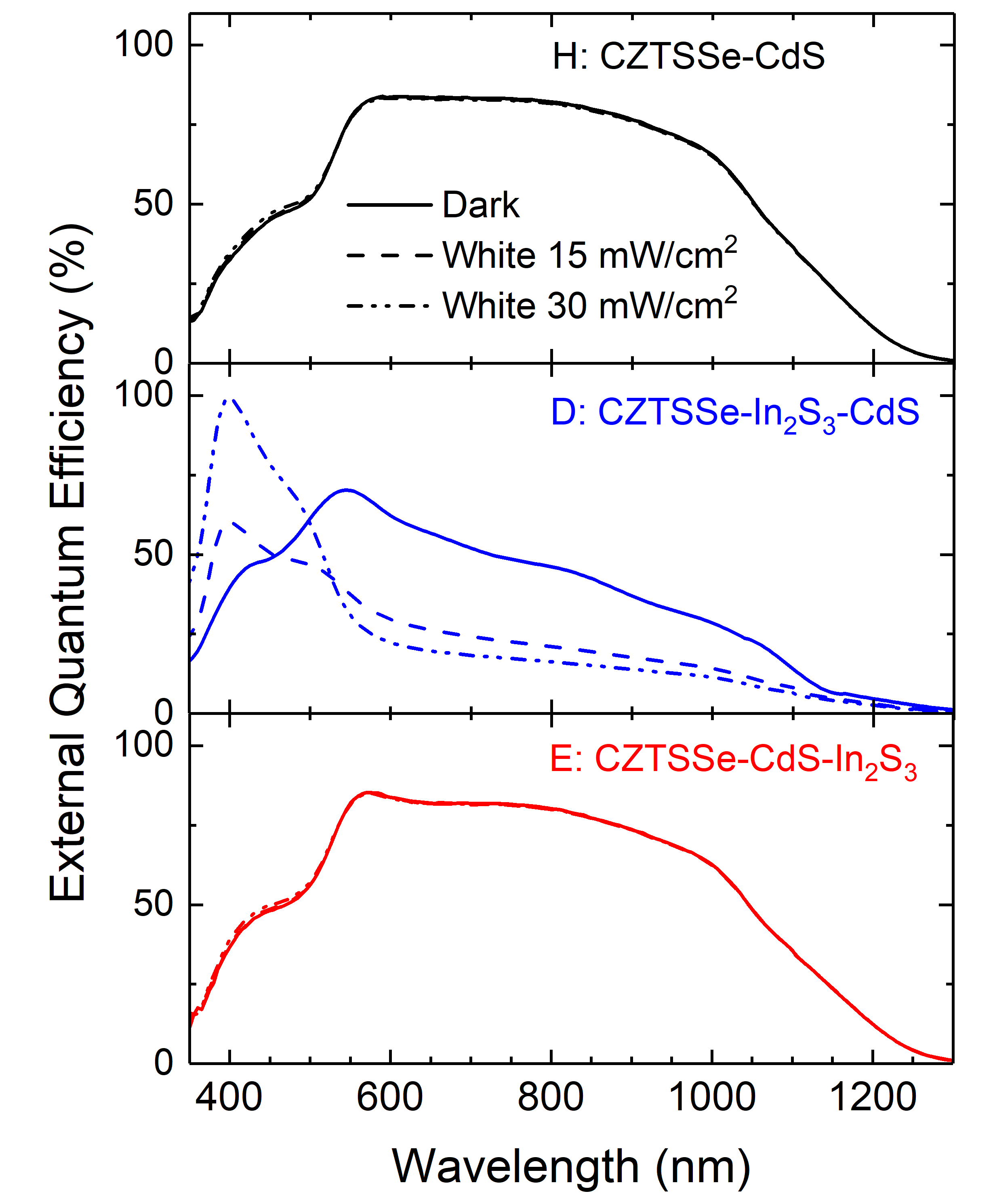

Supplement: Supplementary file 2 — ae3c01622_si_002.zip [file ae3c01622_si_002.zip › EQE_dual buffers_CZTSSe_light_bias.png]

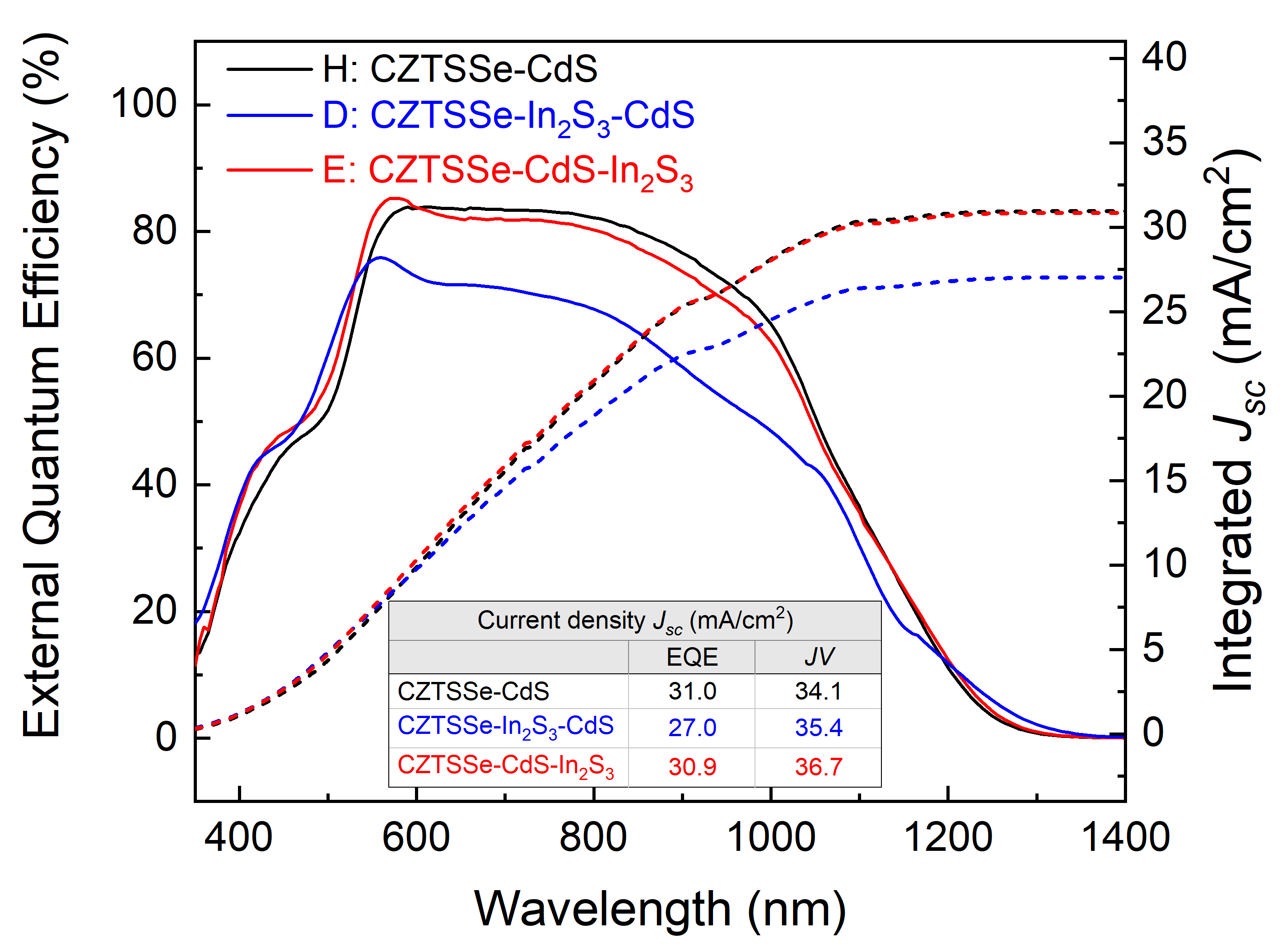

Supplement: Supplementary file 2 — ae3c01622_si_002.zip [file ae3c01622_si_002.zip › EQE_dual_buffers_CZTSSe_integrated_Jsc.png]

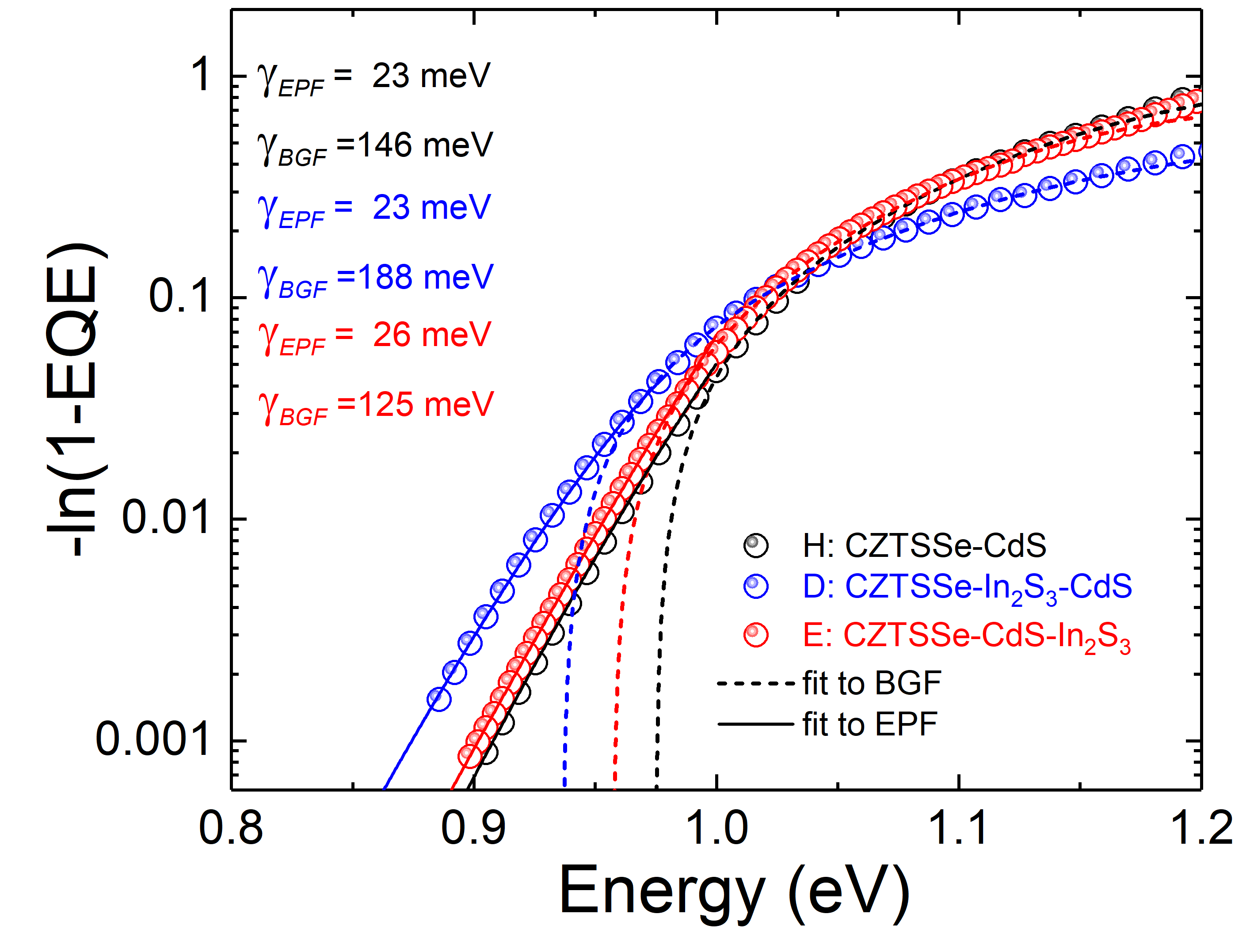

Supplement: Supplementary file 2 — ae3c01622_si_002.zip [file ae3c01622_si_002.zip › EPF_BGF_dual_buffers_CSTSSe_from_EQE.png]

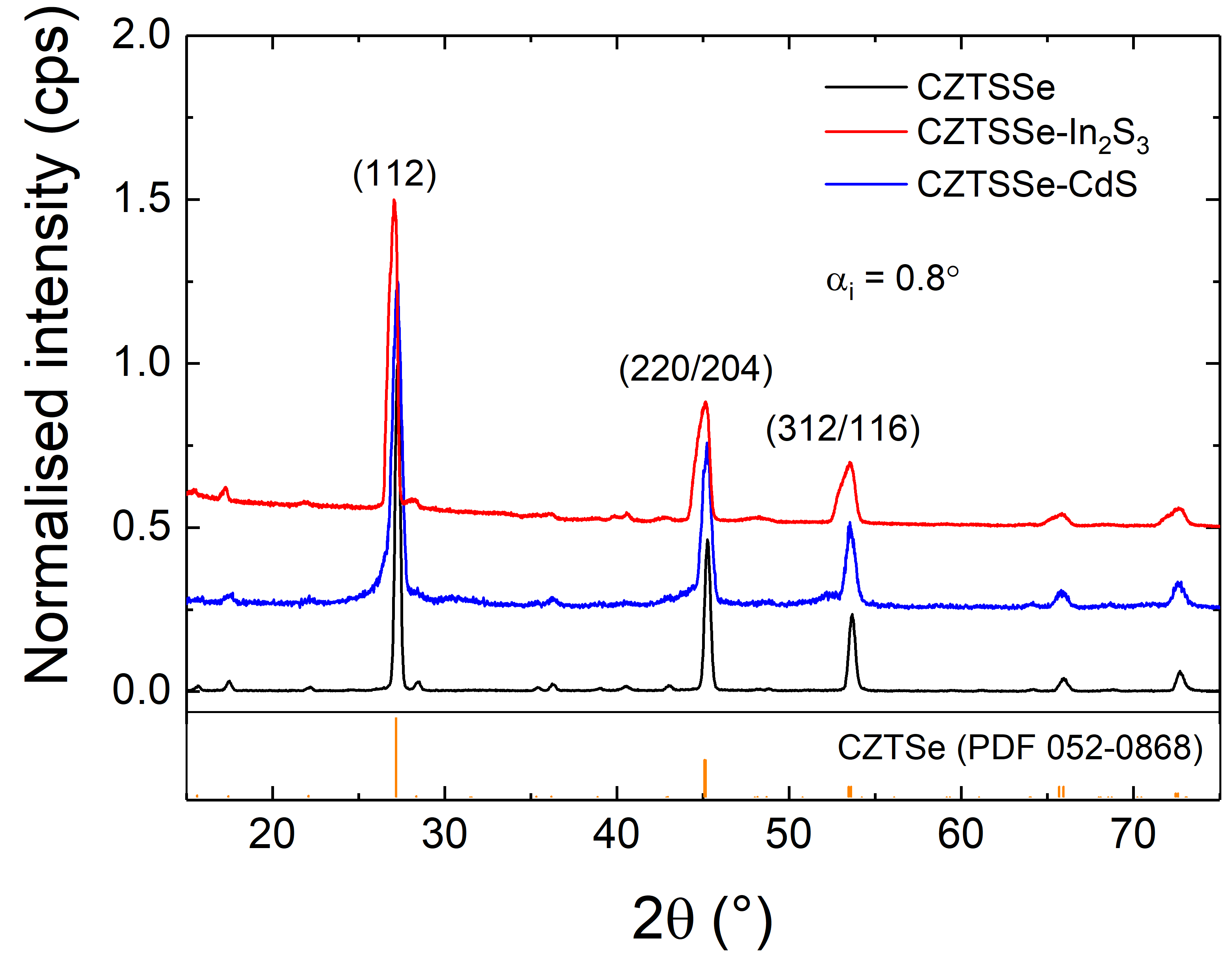

Supplement: Supplementary file 2 — ae3c01622_si_002.zip [file ae3c01622_si_002.zip › XRD_comparison_dual_buffers_CZTSSe.png]

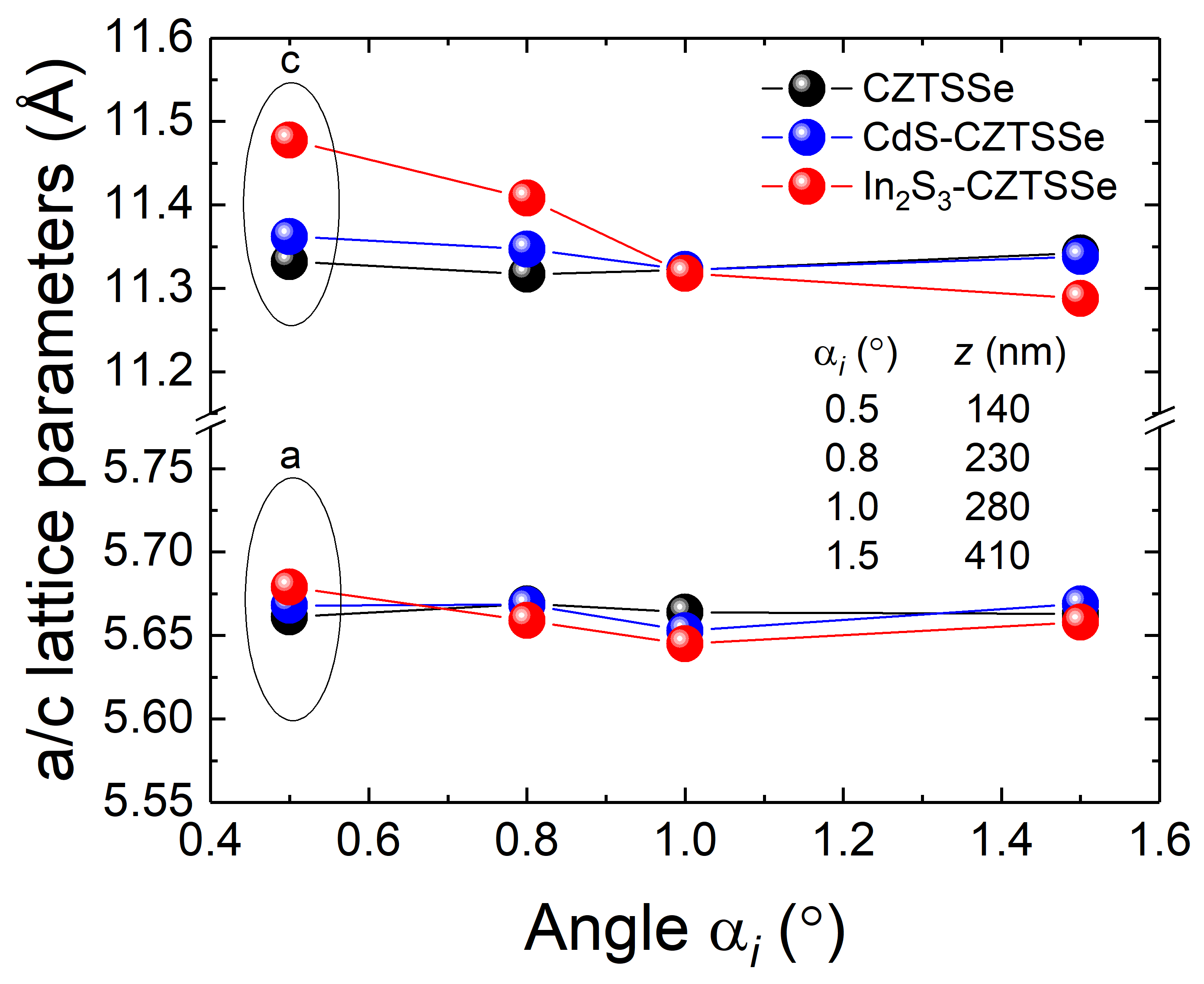

Supplement: Supplementary file 2 — ae3c01622_si_002.zip [file ae3c01622_si_002.zip › XRD_lattice_comparison_dual_buffers_CZTSSe.png]

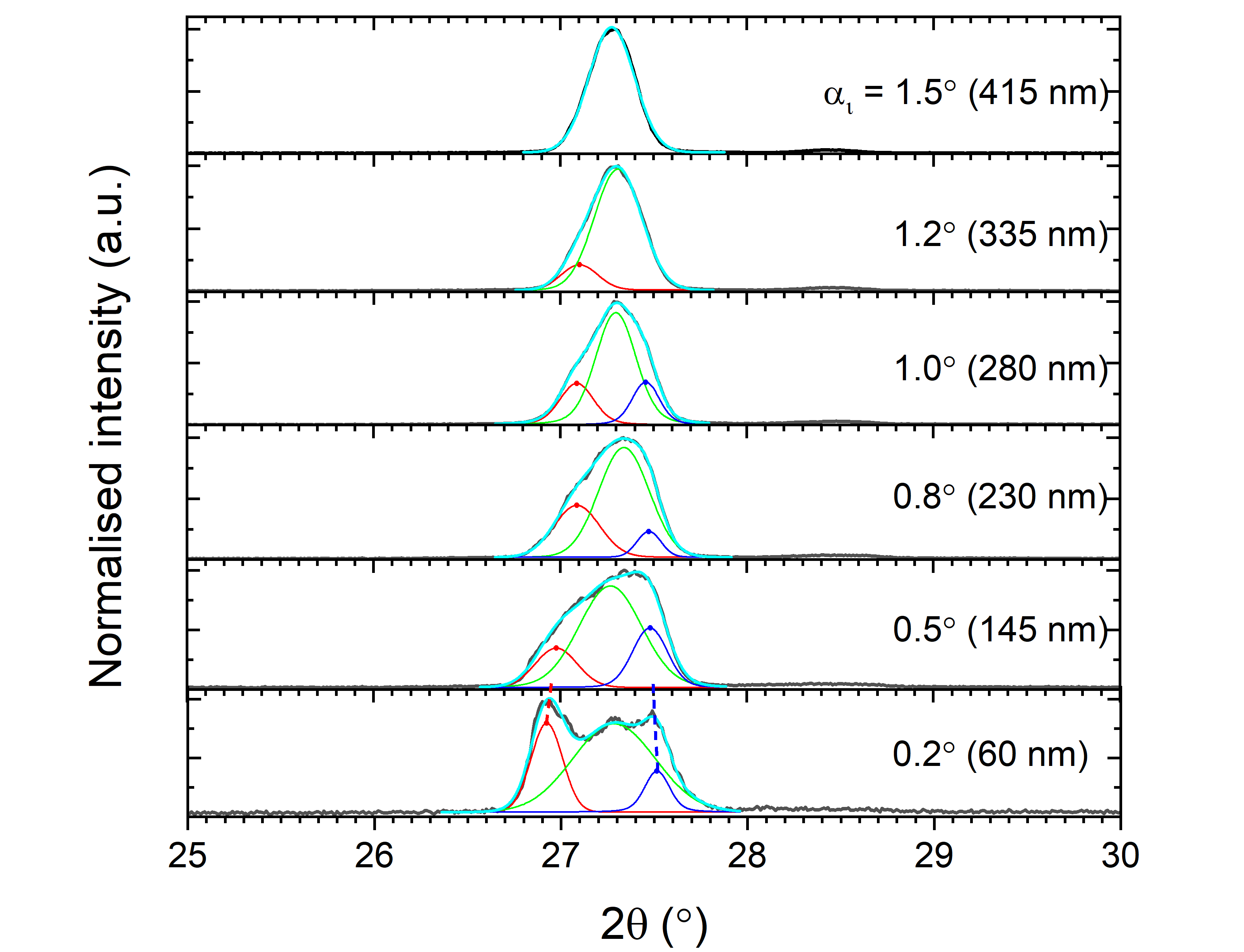

Supplement: Supplementary file 2 — ae3c01622_si_002.zip [file ae3c01622_si_002.zip › CZTSSe_In2S3_peak_fit_GIXRD.png]

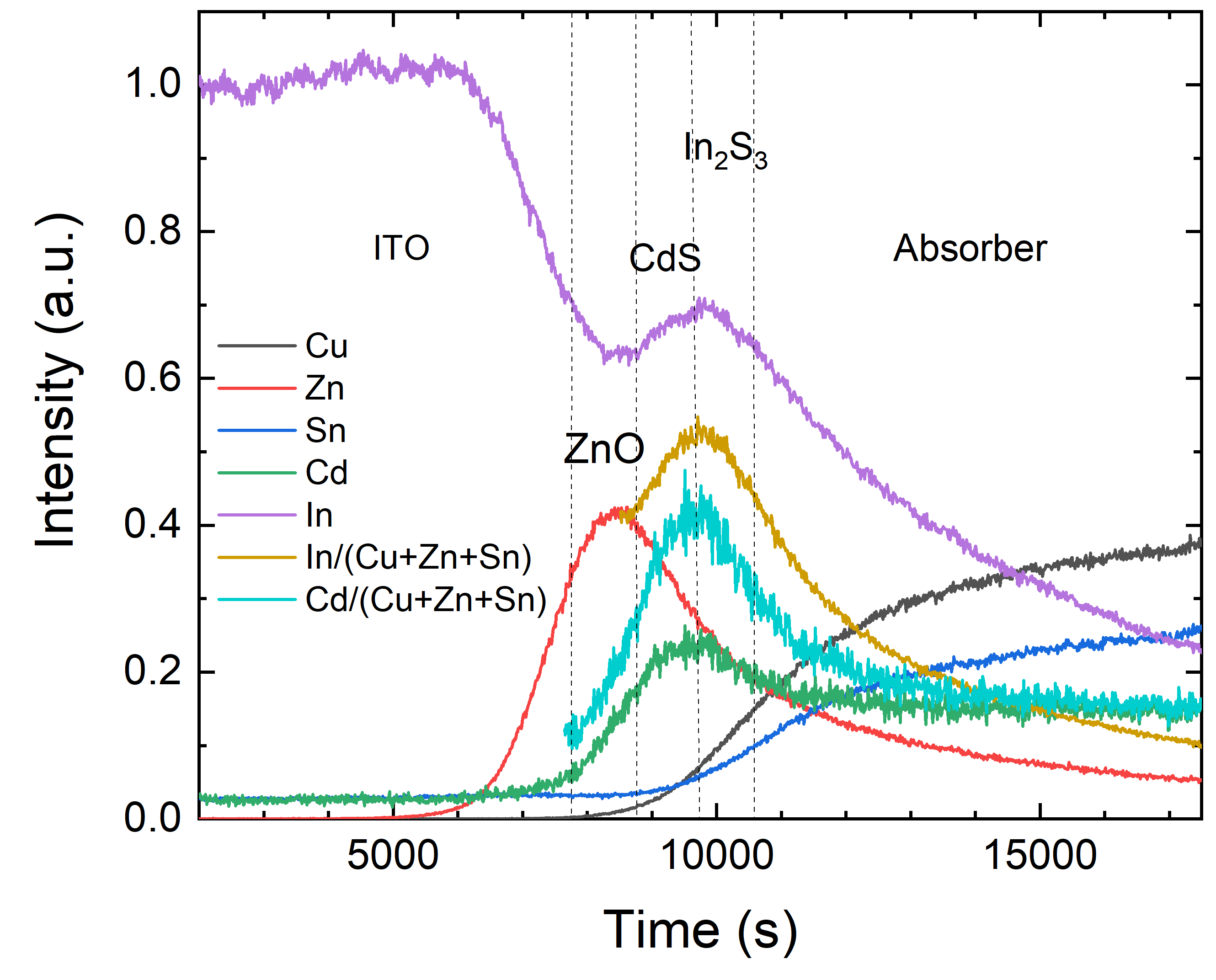

Supplement: Supplementary file 2 — ae3c01622_si_002.zip [file ae3c01622_si_002.zip › SIMS_CdS_In2S3_CZTSSe.png]

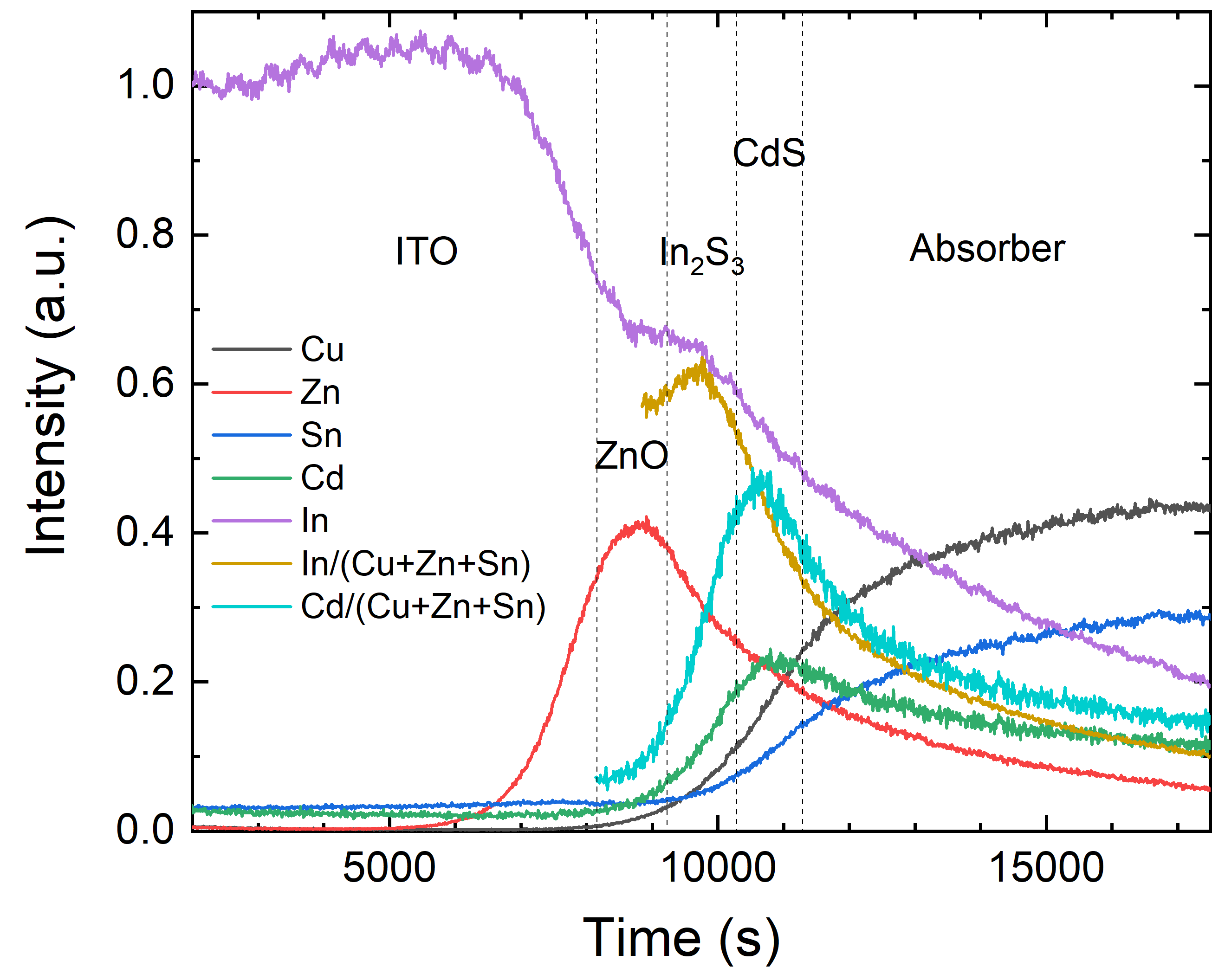

Supplement: Supplementary file 2 — ae3c01622_si_002.zip [file ae3c01622_si_002.zip › SIMS_In2S3_CdS_CZTSSe.png]

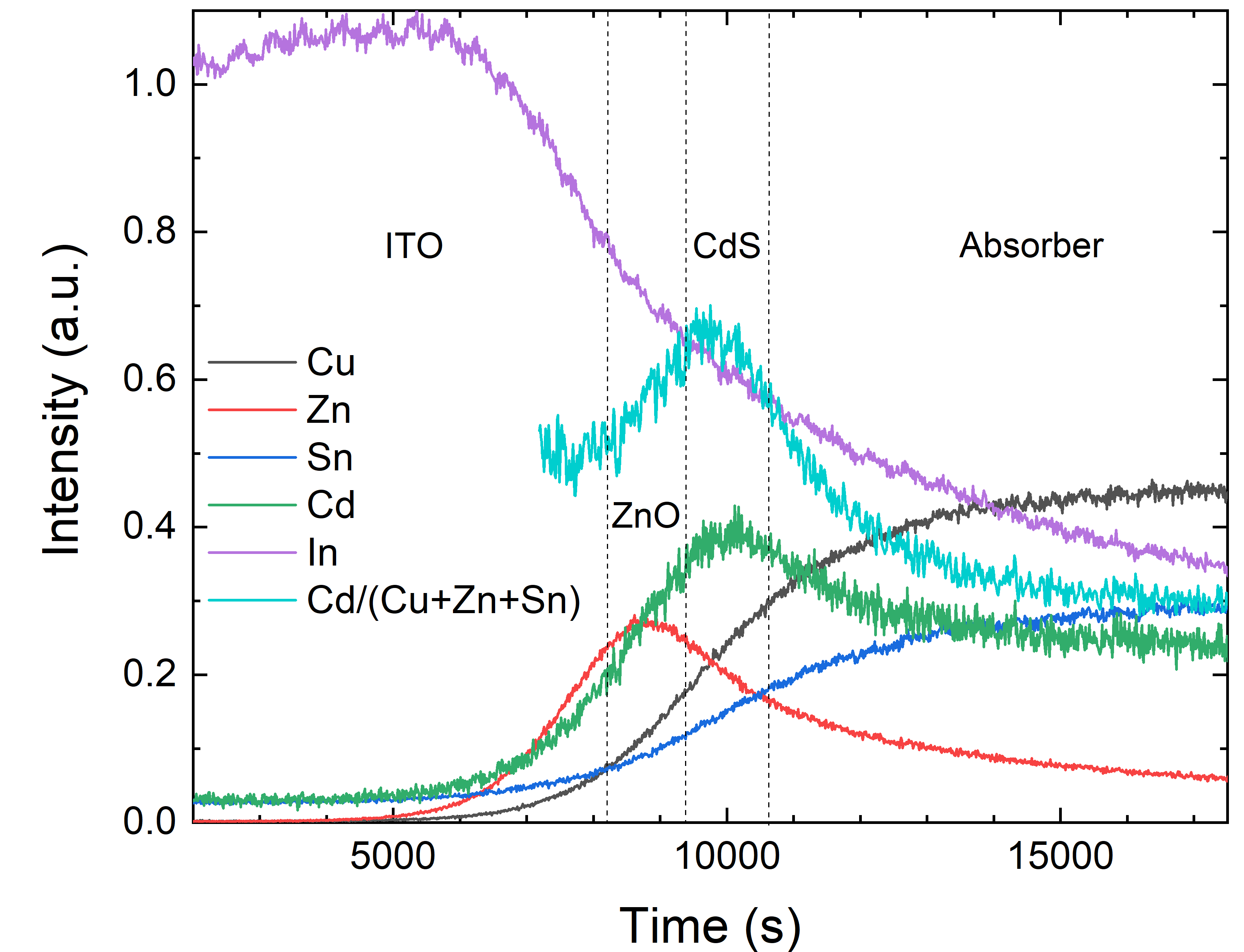

Supplement: Supplementary file 2 — ae3c01622_si_002.zip [file ae3c01622_si_002.zip › SIMS_CdS_CZTSSe.png]

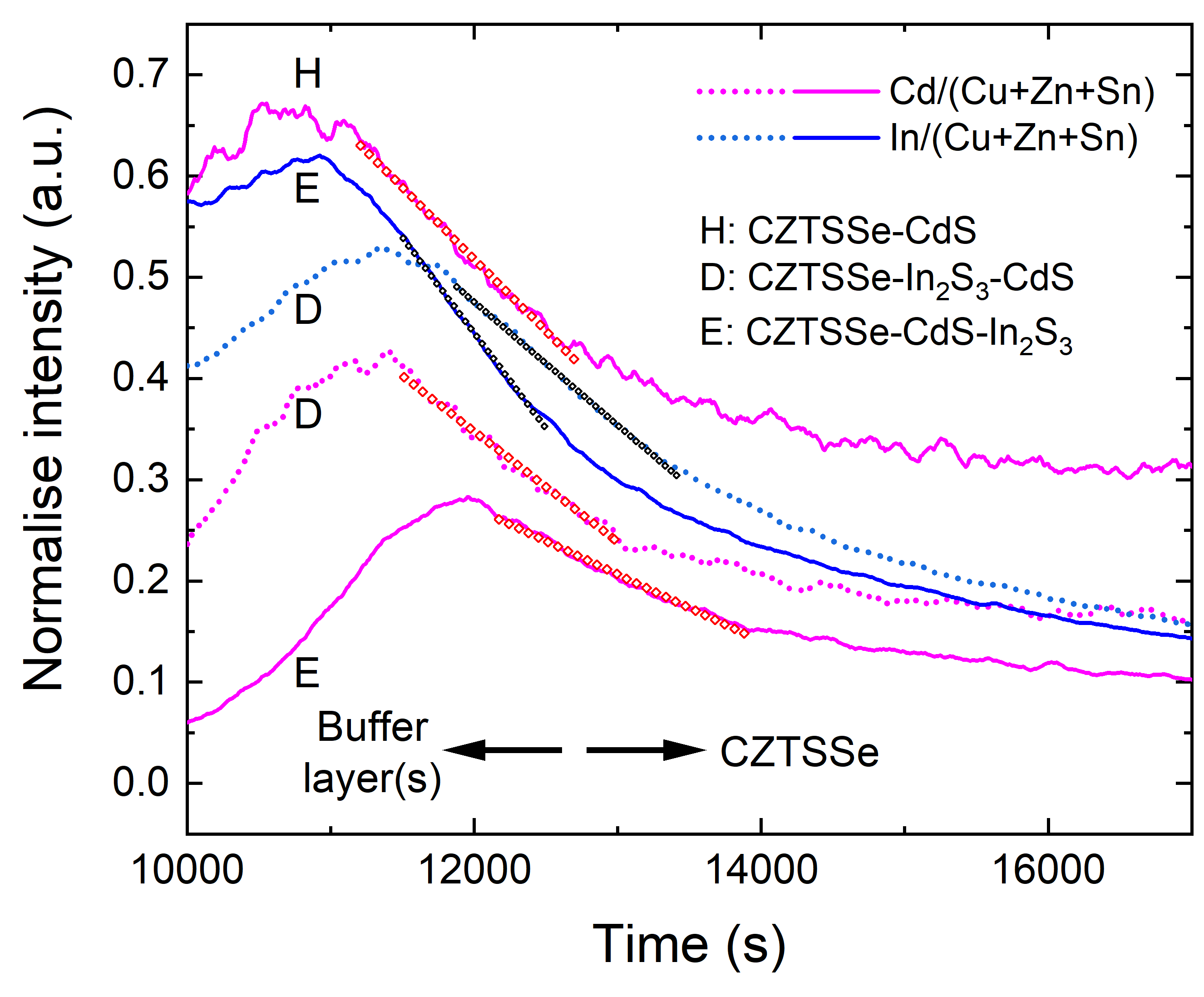

Supplement: Supplementary file 2 — ae3c01622_si_002.zip [file ae3c01622_si_002.zip › SIMS_Cd_In_decay_dual_buffers_H_D_E.png]

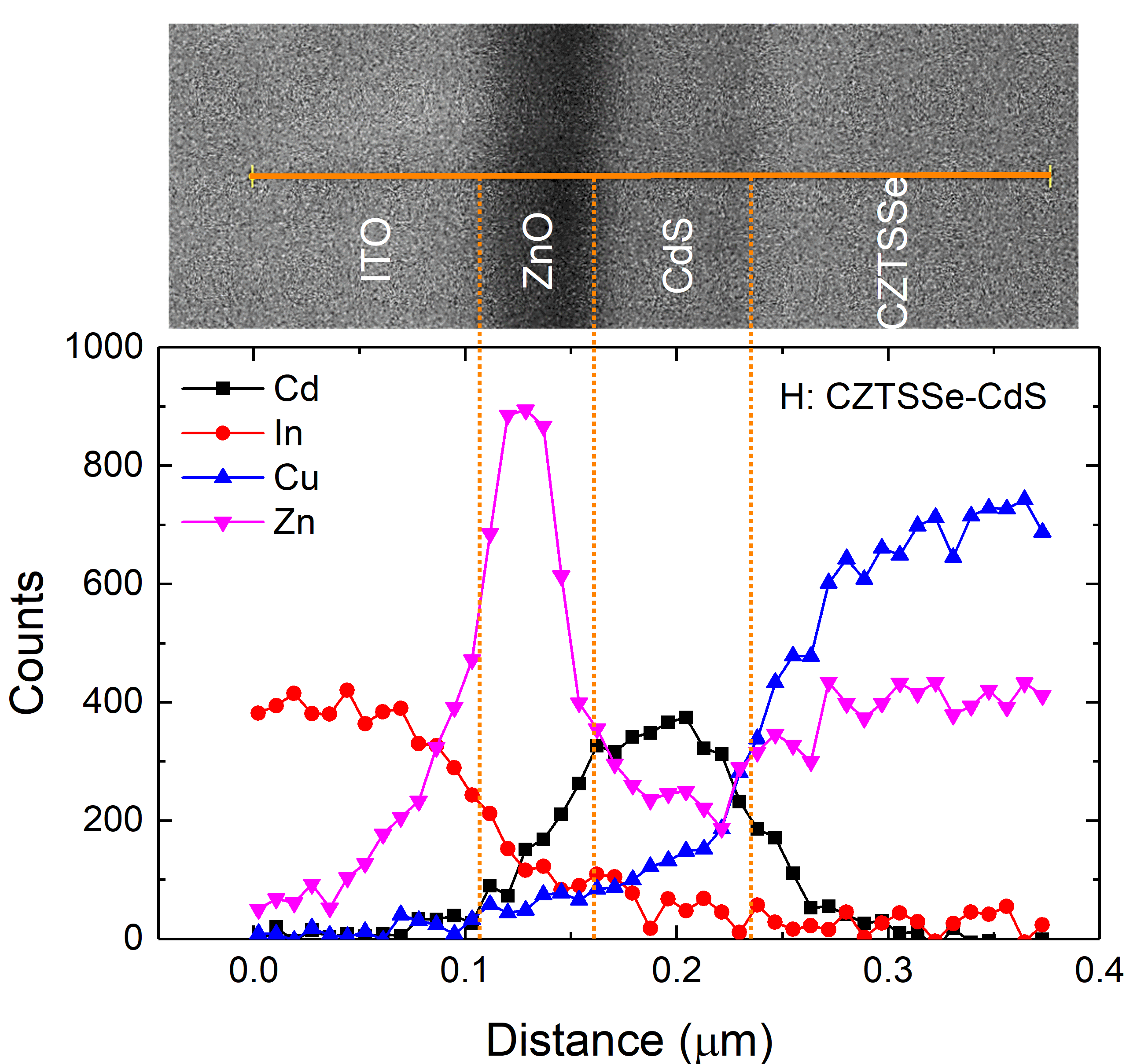

Supplement: Supplementary file 2 — ae3c01622_si_002.zip [file ae3c01622_si_002.zip › SEM_EDS_linescan_H_CZTSSe_CdS.png]

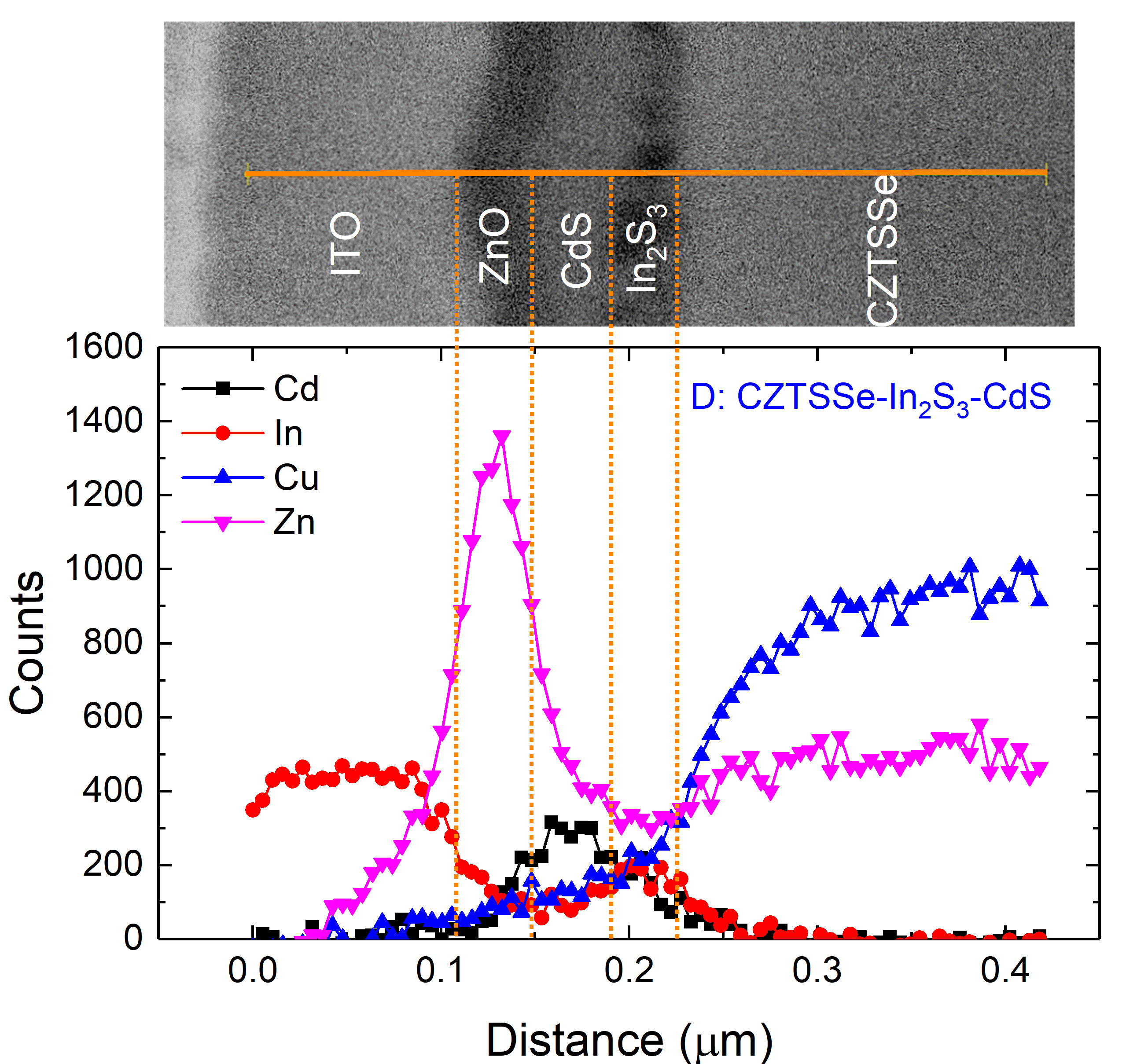

Supplement: Supplementary file 2 — ae3c01622_si_002.zip [file ae3c01622_si_002.zip › SEM_EDS_linescan_D_CZTSSe_In2S3_CdS.png]

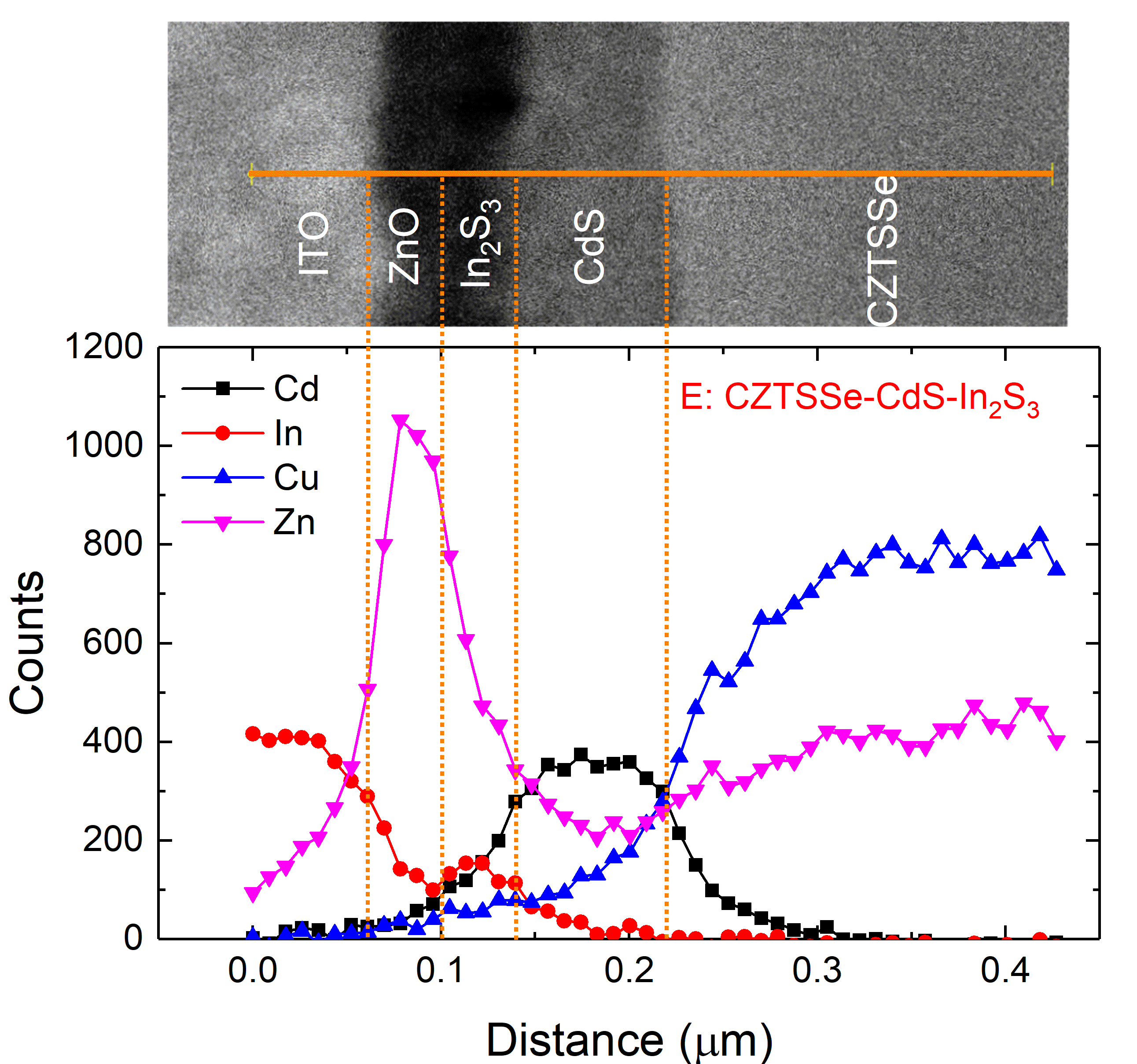

Supplement: Supplementary file 2 — ae3c01622_si_002.zip [file ae3c01622_si_002.zip › SEM_EDS_linescan_E_CZTSSe_CdS_In2S3.png]

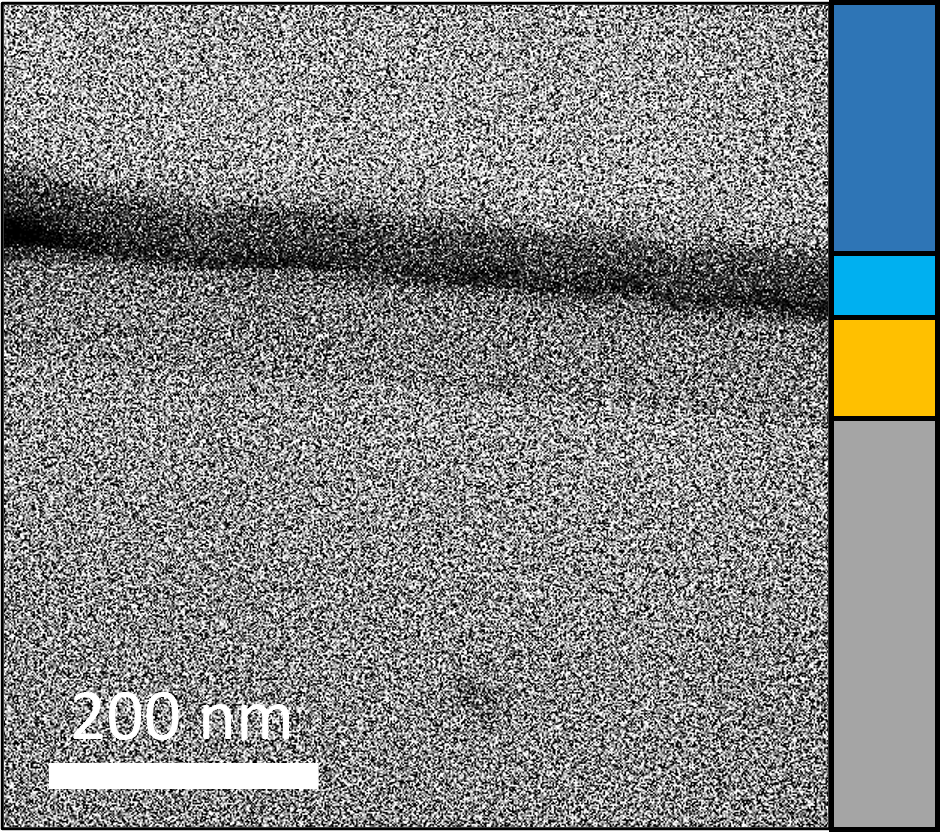

Supplement: Supplementary file 2 — ae3c01622_si_002.zip [file ae3c01622_si_002.zip › BSE_EDS_190314H_with_key.png]

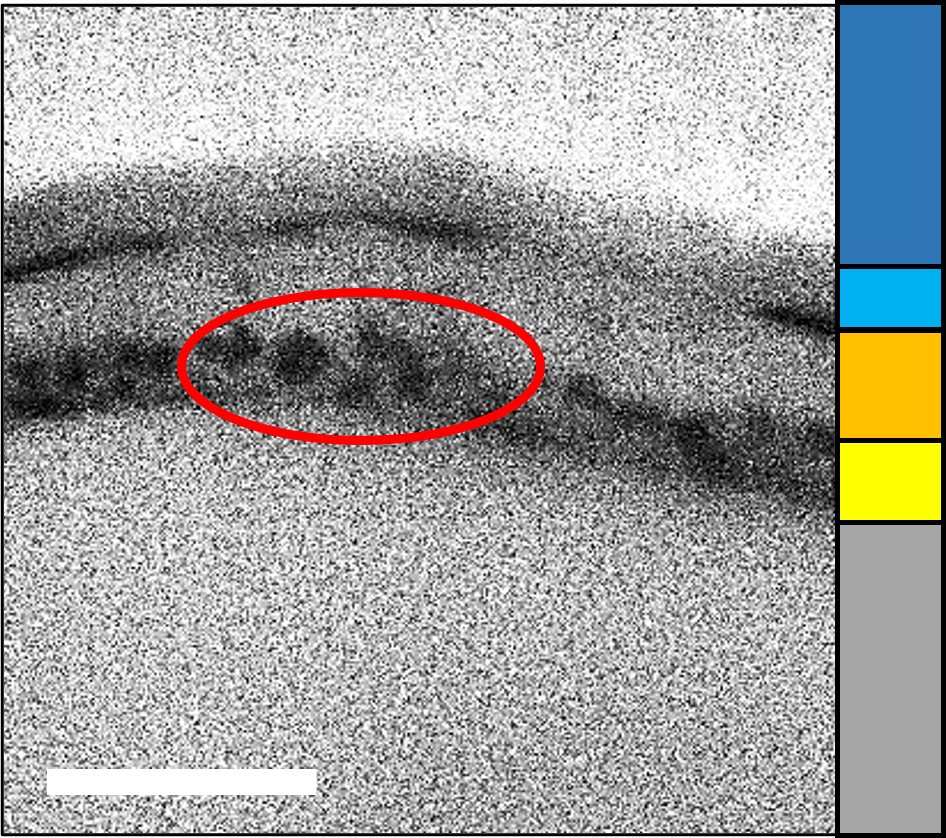

Supplement: Supplementary file 2 — ae3c01622_si_002.zip [file ae3c01622_si_002.zip › BSE_EDS_190314D_highlighted_with_key.png]

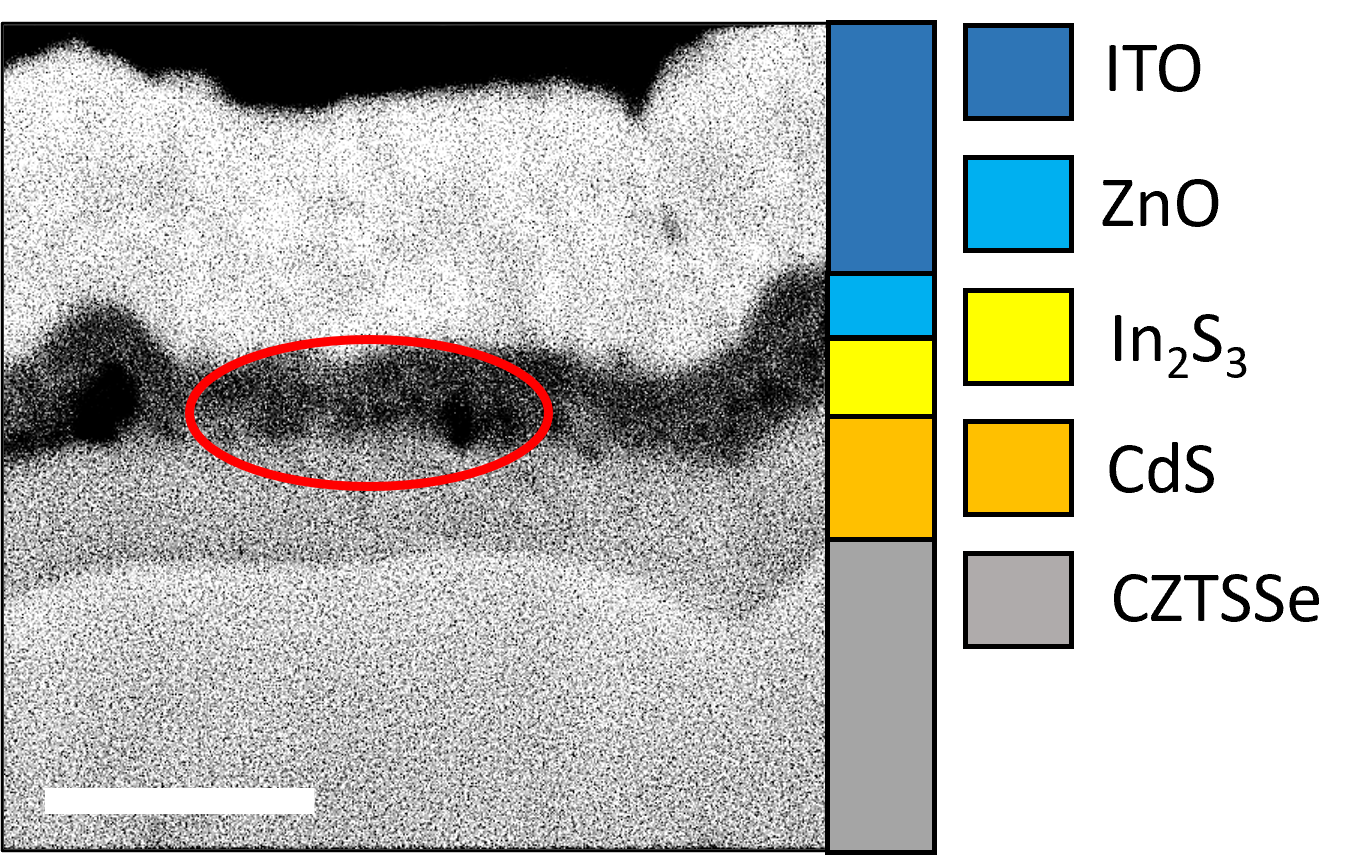

Supplement: Supplementary file 2 — ae3c01622_si_002.zip [file ae3c01622_si_002.zip › BSE_EDS_190314E_highlighted_with_key.png]

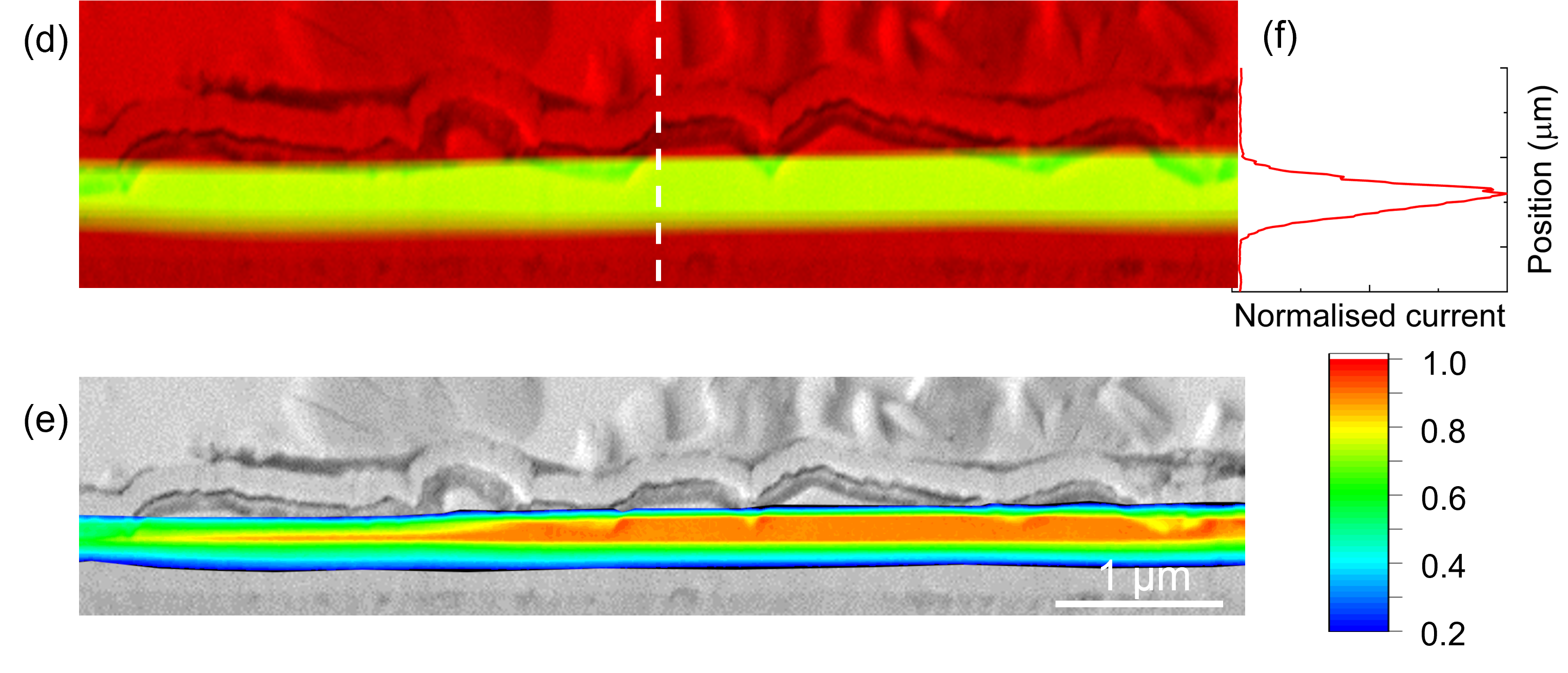

Supplement: Supplementary file 2 — ae3c01622_si_002.zip [file ae3c01622_si_002.zip › EBIC_combined_190314E.png]

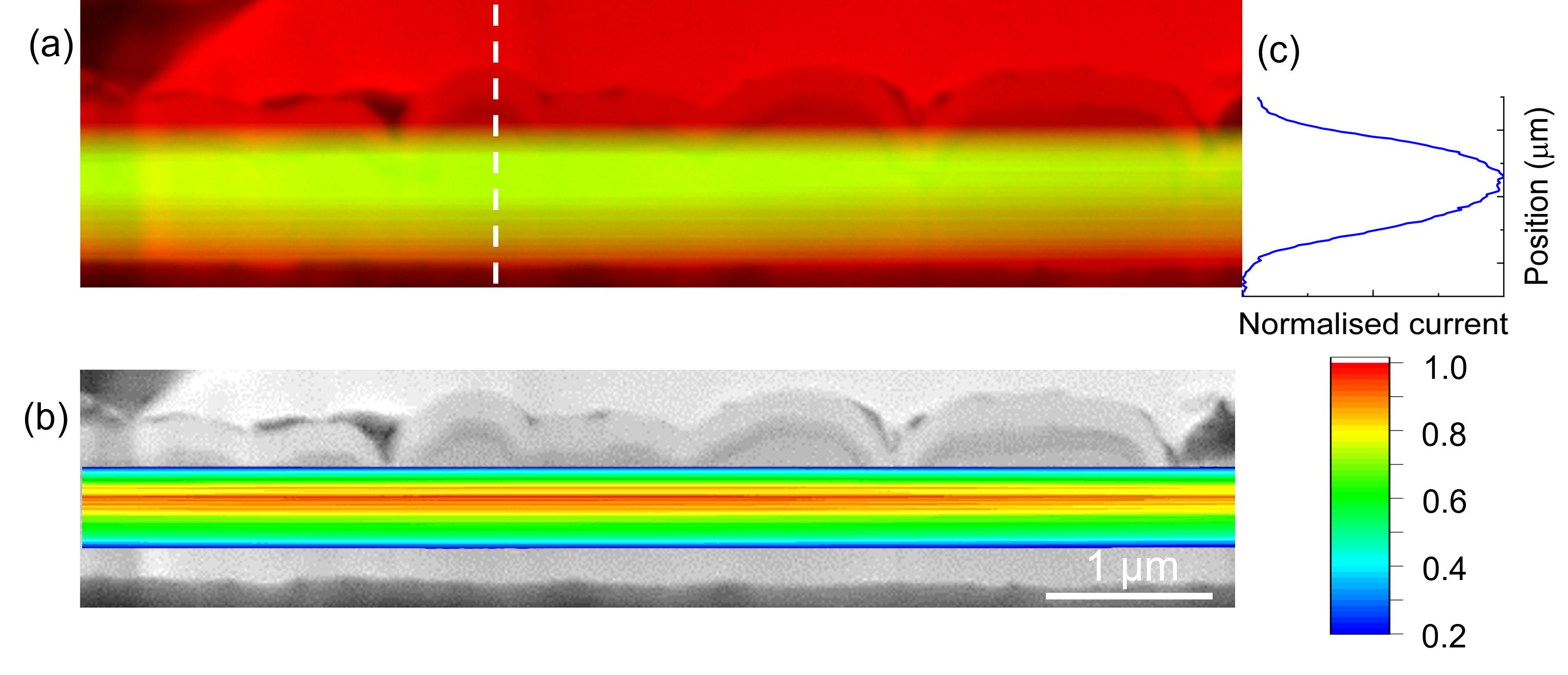

Supplement: Supplementary file 2 — ae3c01622_si_002.zip [file ae3c01622_si_002.zip › EBIC_combined_190314D.png]

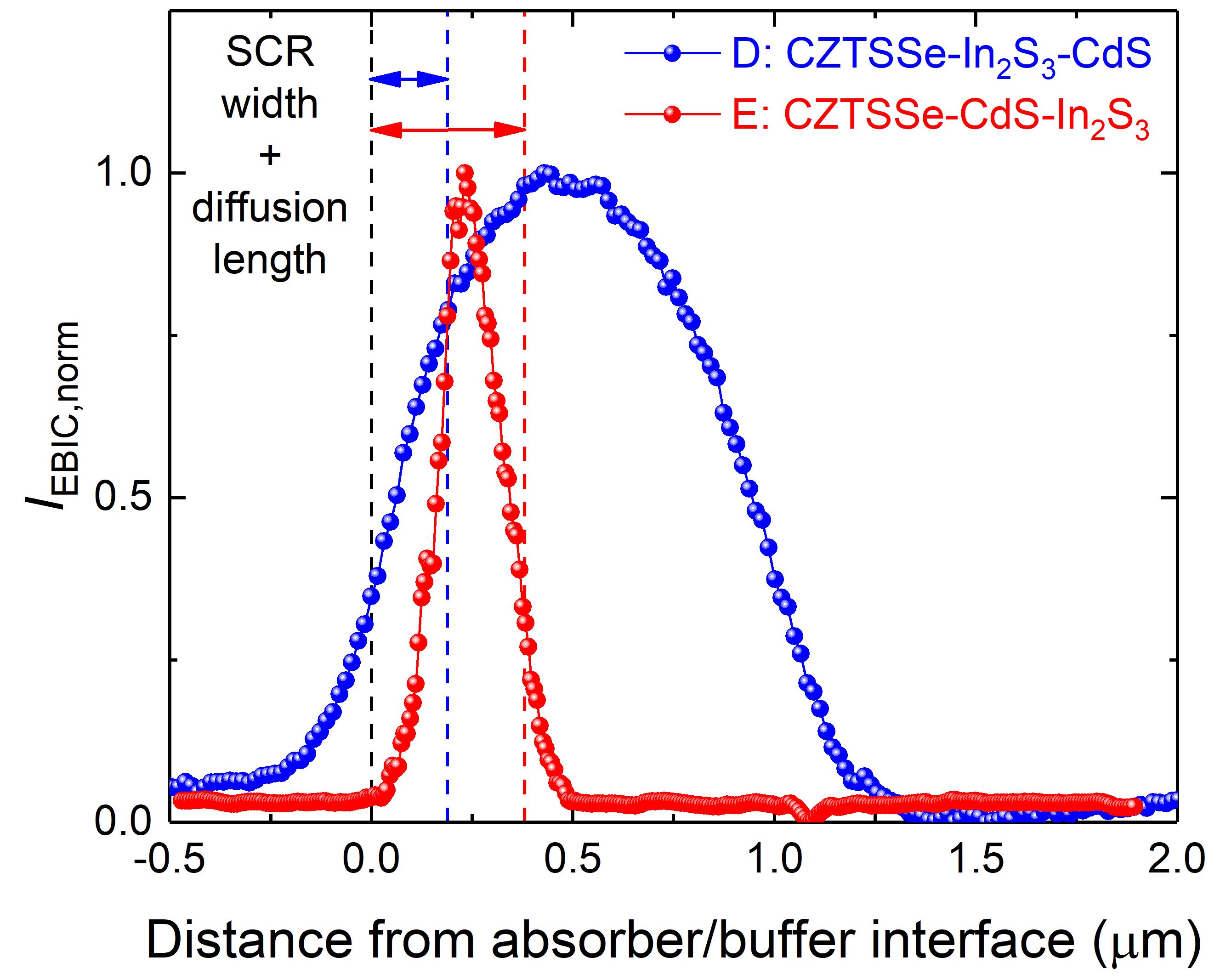

Supplement: Supplementary file 2 — ae3c01622_si_002.zip [file ae3c01622_si_002.zip › EBIC_linescan_comparison_effective_diffusion_length.png]

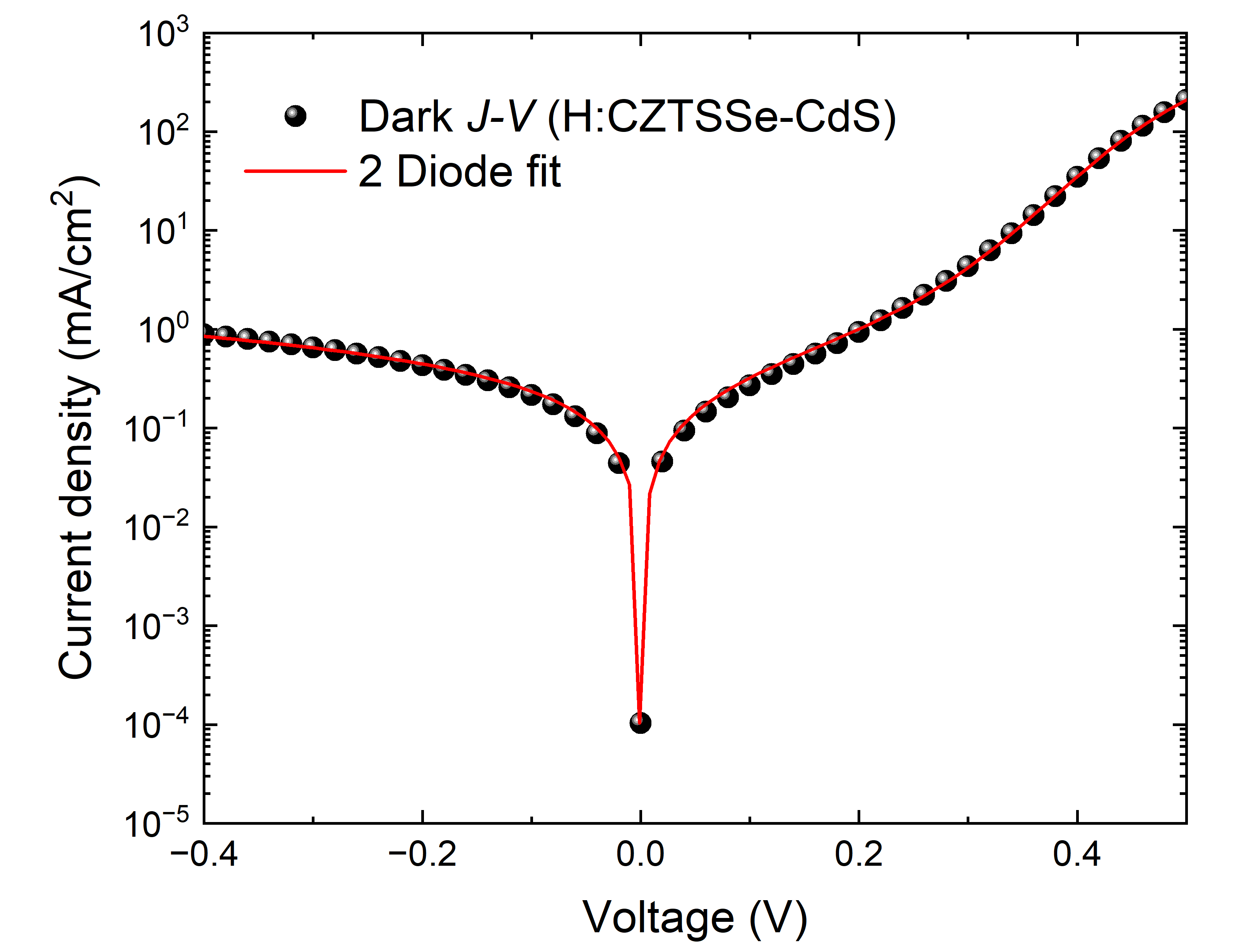

Supplement: Supplementary file 2 — ae3c01622_si_002.zip [file ae3c01622_si_002.zip › 2_diode_fit_dark_JV_190314H.png]

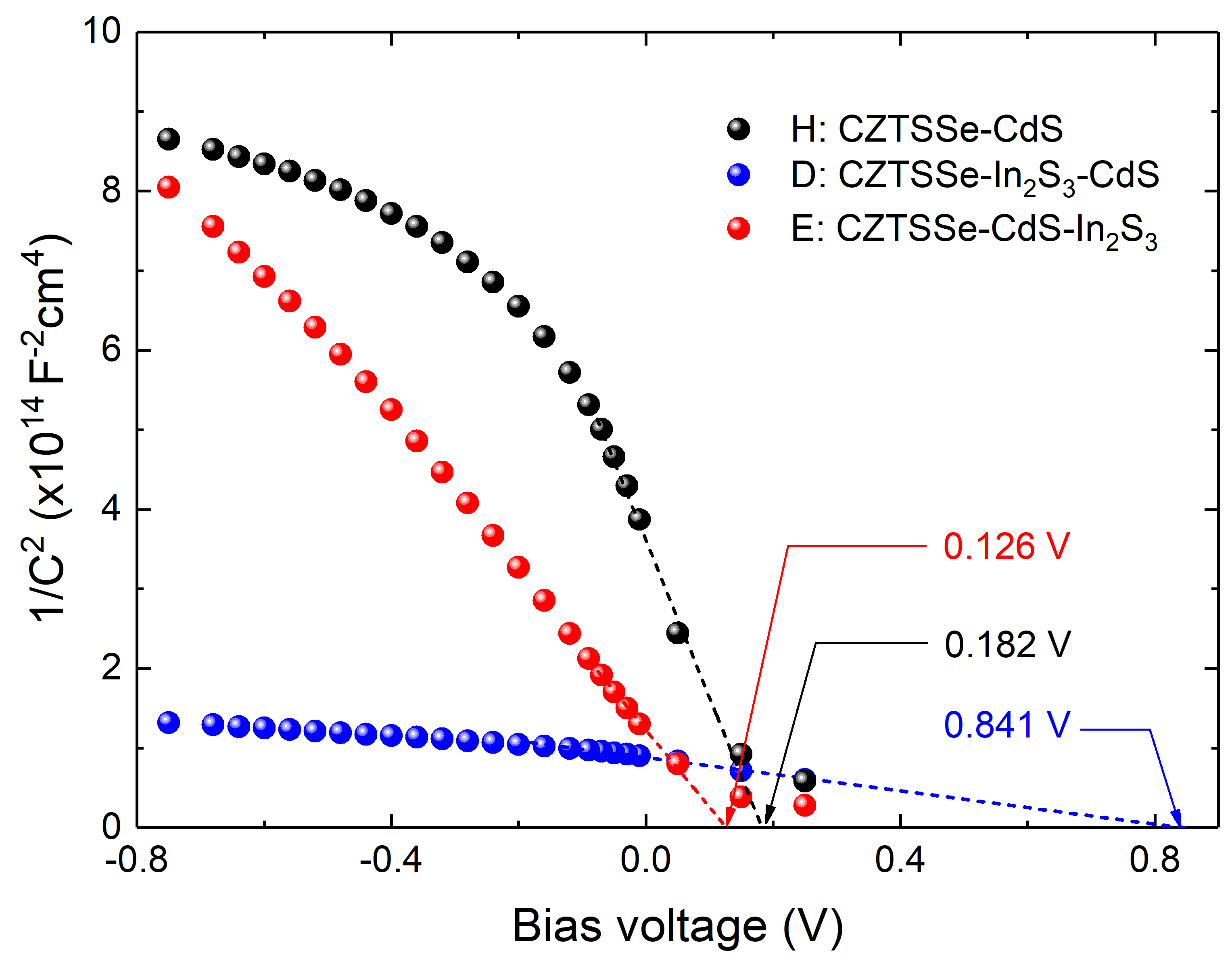

Supplement: Supplementary file 2 — ae3c01622_si_002.zip [file ae3c01622_si_002.zip › Vbi_dual_buffers_CZTSSe.png]

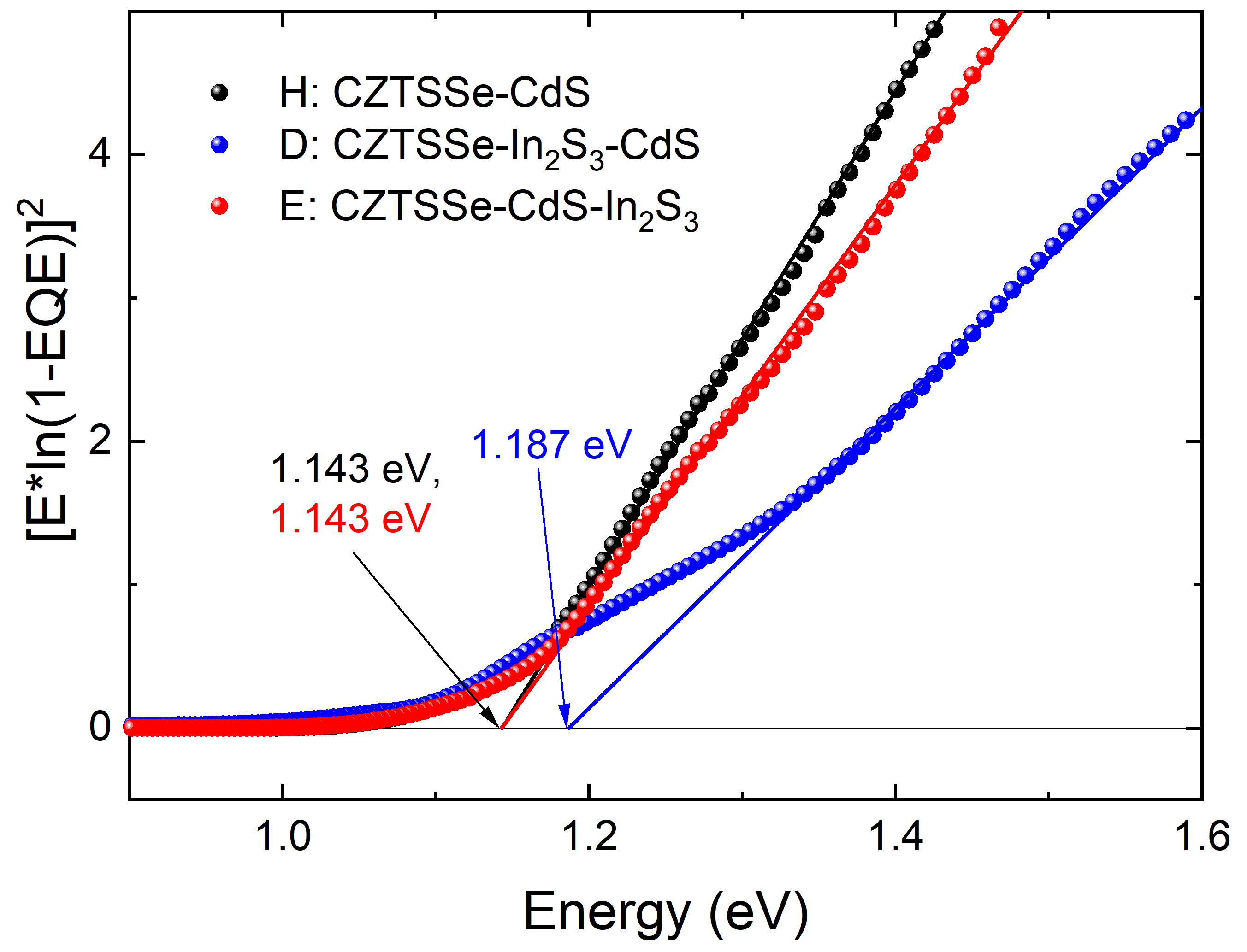

Supplement: Supplementary file 2 — ae3c01622_si_002.zip [file ae3c01622_si_002.zip › Bandgap_EQE_dual_buffer_CZTSSe.png]

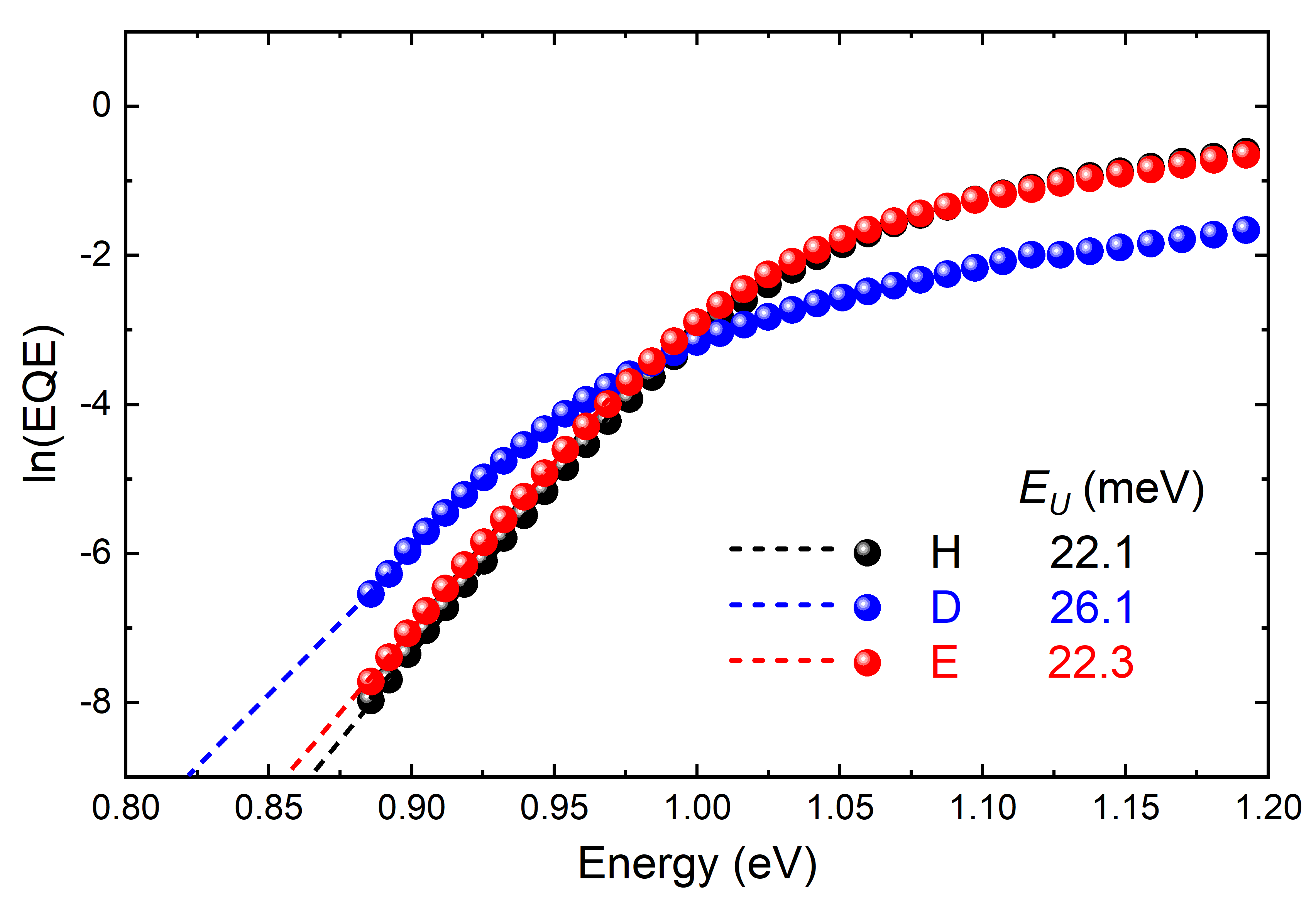

Supplement: Supplementary file 2 — ae3c01622_si_002.zip [file ae3c01622_si_002.zip › Urbach_energy_dual_buffer_CZTSSe.png]

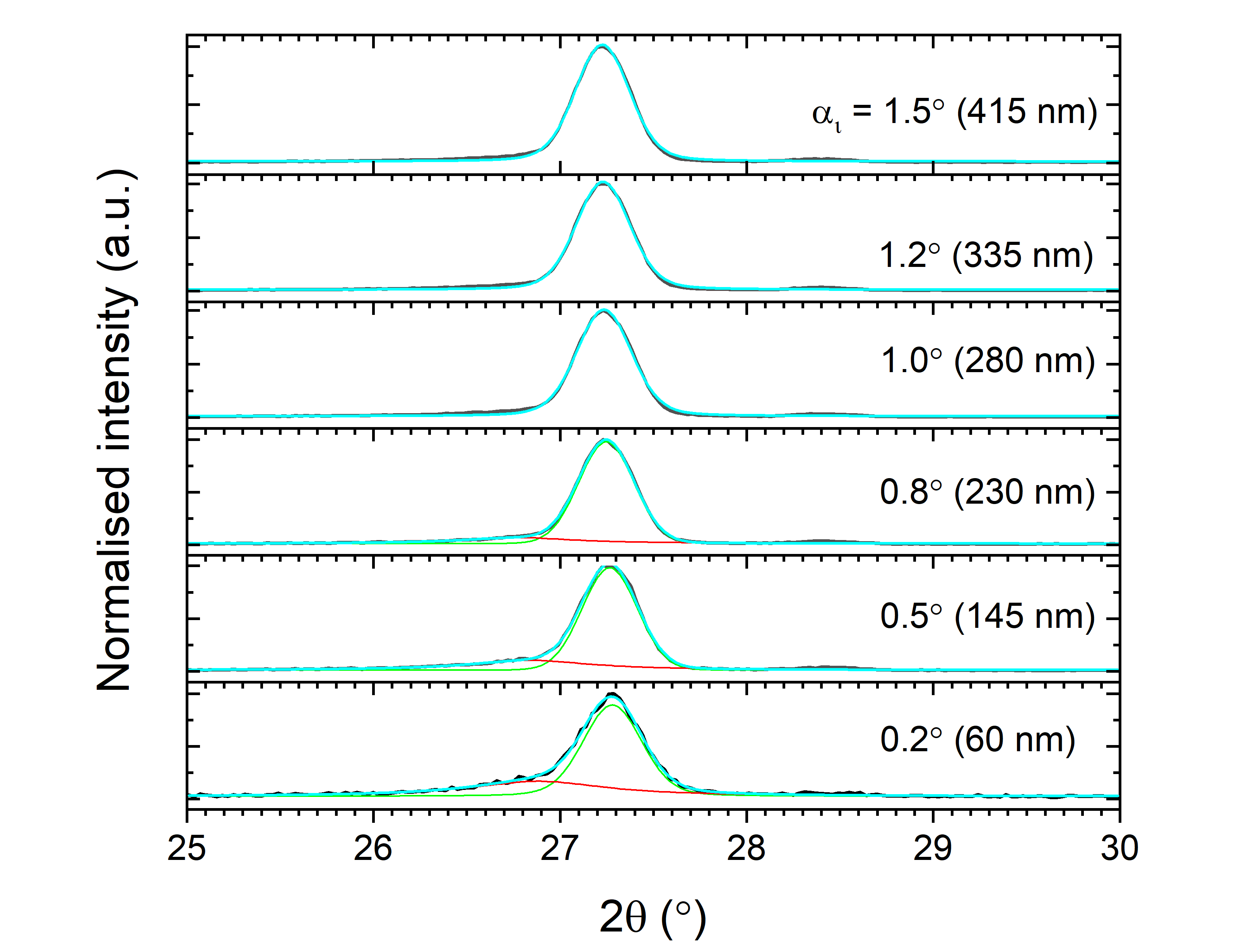

Supplement: Supplementary file 2 — ae3c01622_si_002.zip [file ae3c01622_si_002.zip › CZTSSe_CdS_peak_fit_GIXRD.png]

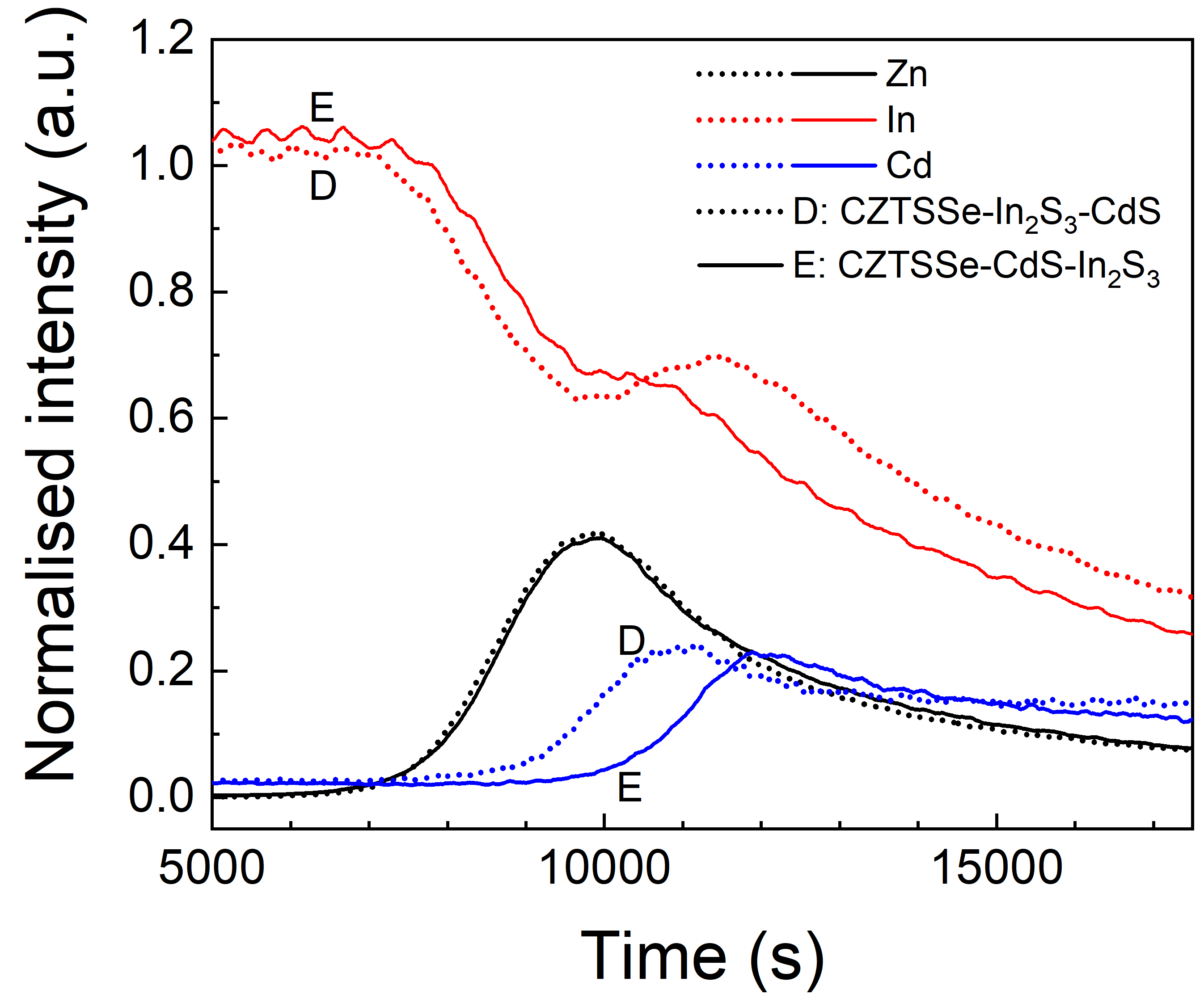

Supplement: Supplementary file 2 — ae3c01622_si_002.zip [file ae3c01622_si_002.zip › SIMS_Zn_peak_overlap_D_and_E.png]

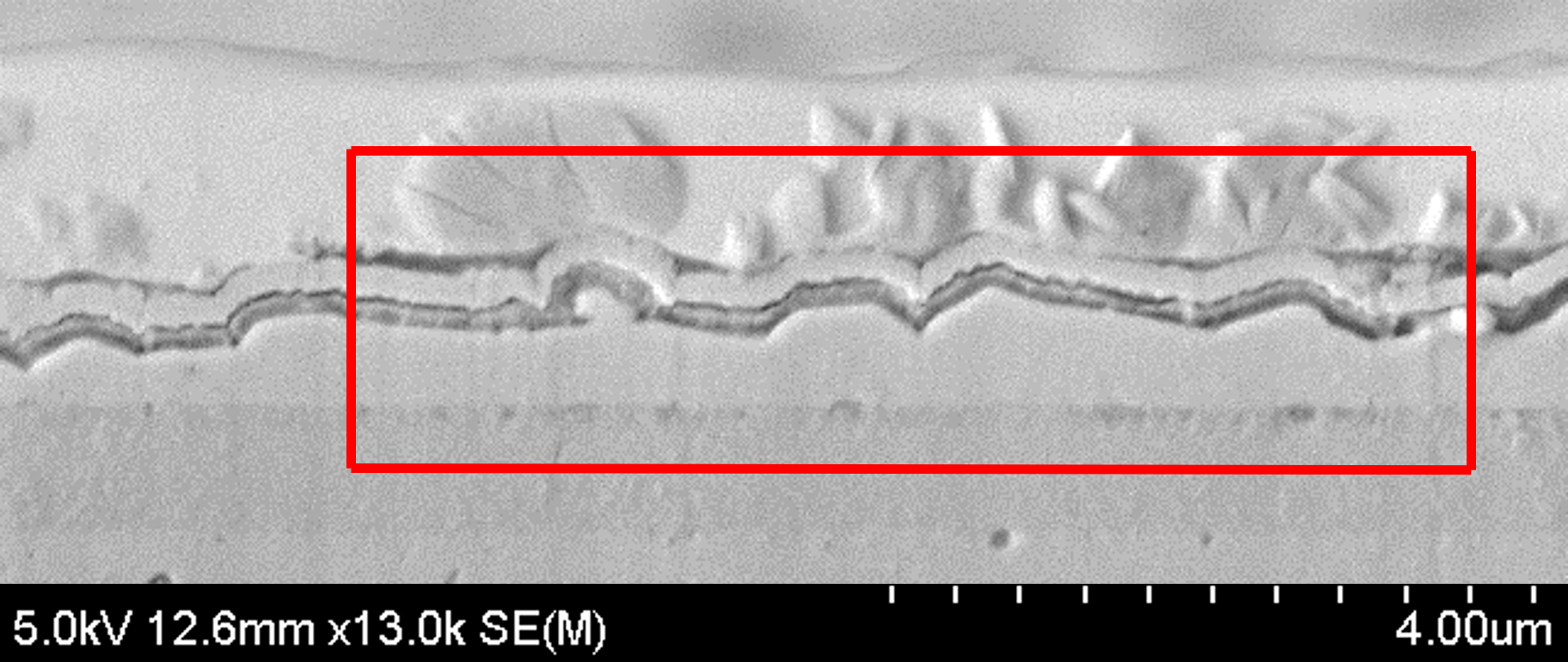

Supplement: Supplementary file 2 — ae3c01622_si_002.zip [file ae3c01622_si_002.zip › Durham_FIB_190314E_4_micron.png]

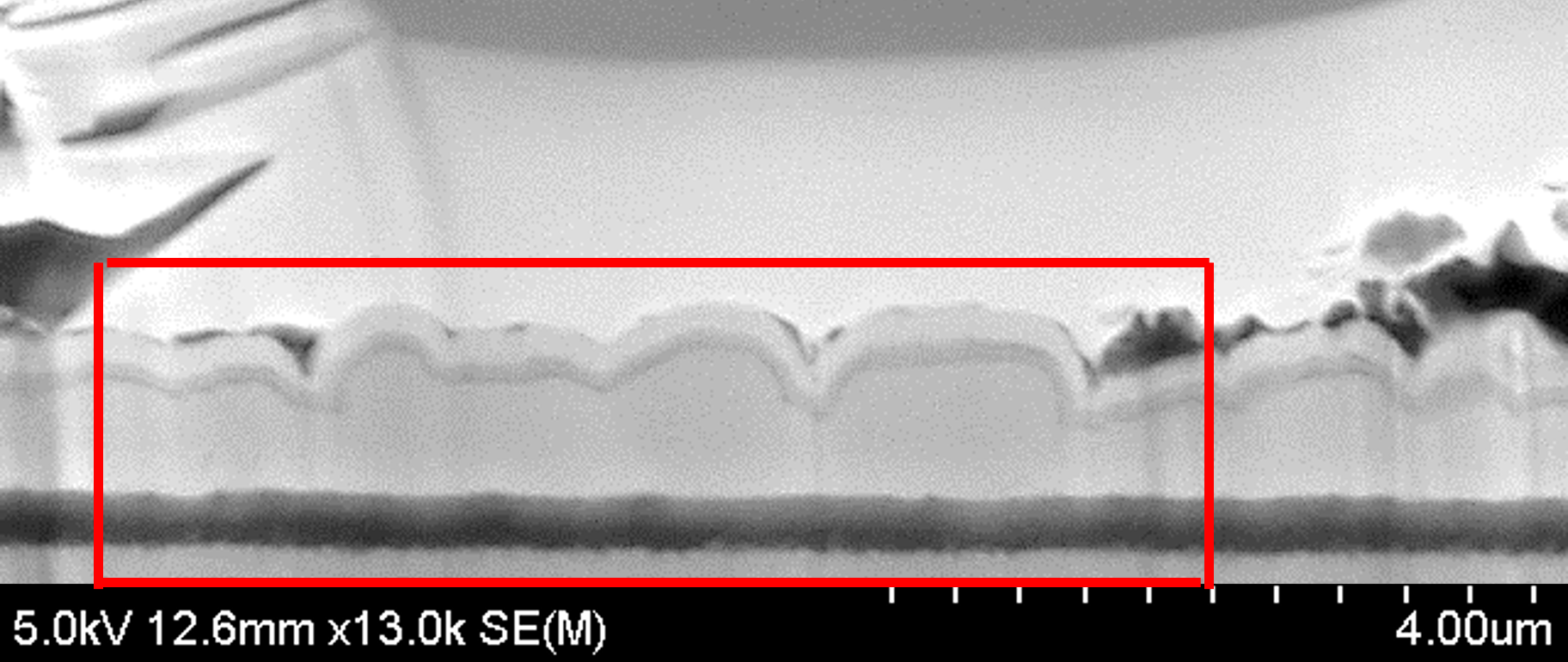

Supplement: Supplementary file 2 — ae3c01622_si_002.zip [file ae3c01622_si_002.zip › Durham_FIB_190314D_4_micron.png]

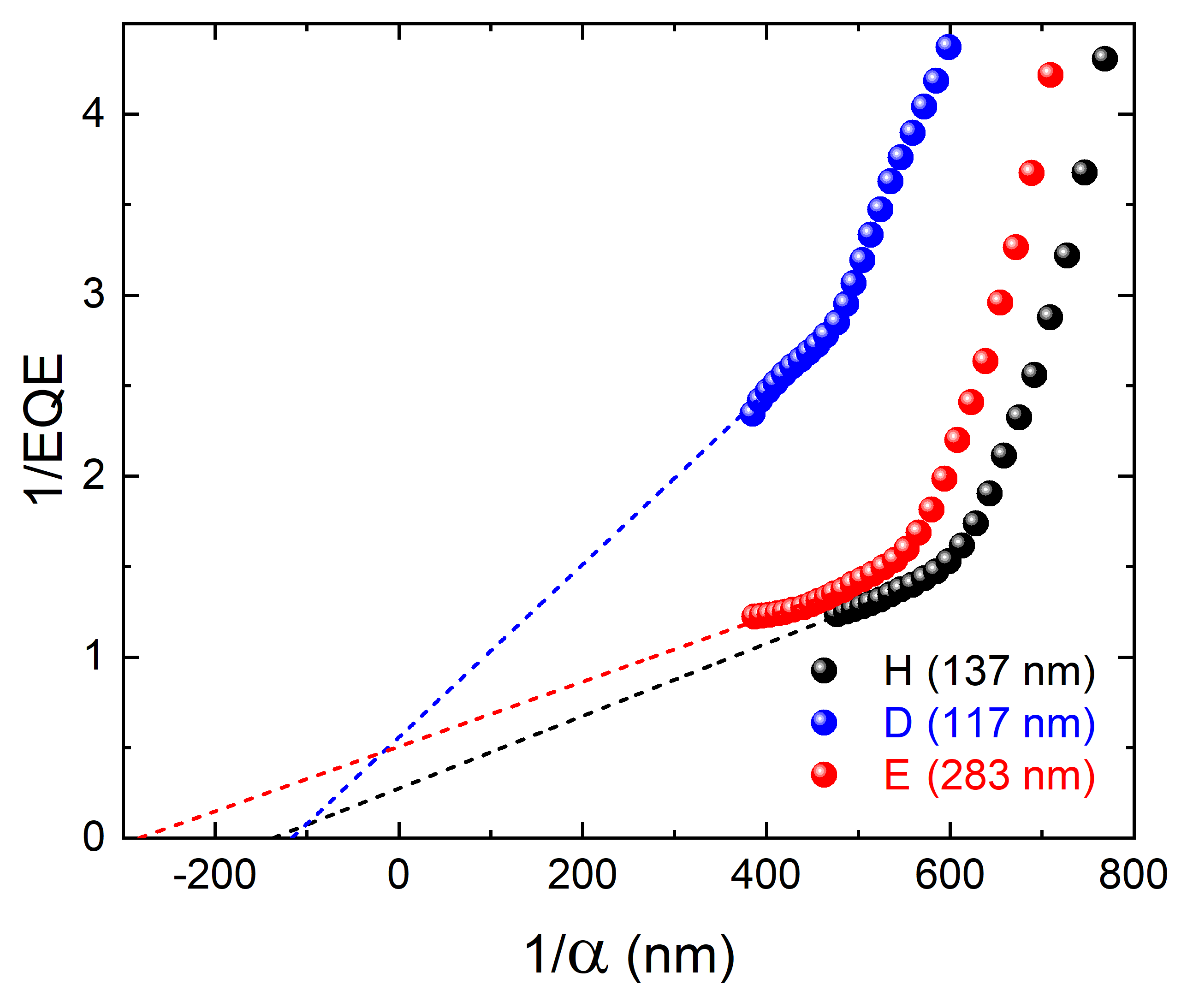

Supplement: Supplementary file 2 — ae3c01622_si_002.zip [file ae3c01622_si_002.zip › Dual_buffer_CZTSSe_difusion_length.png]

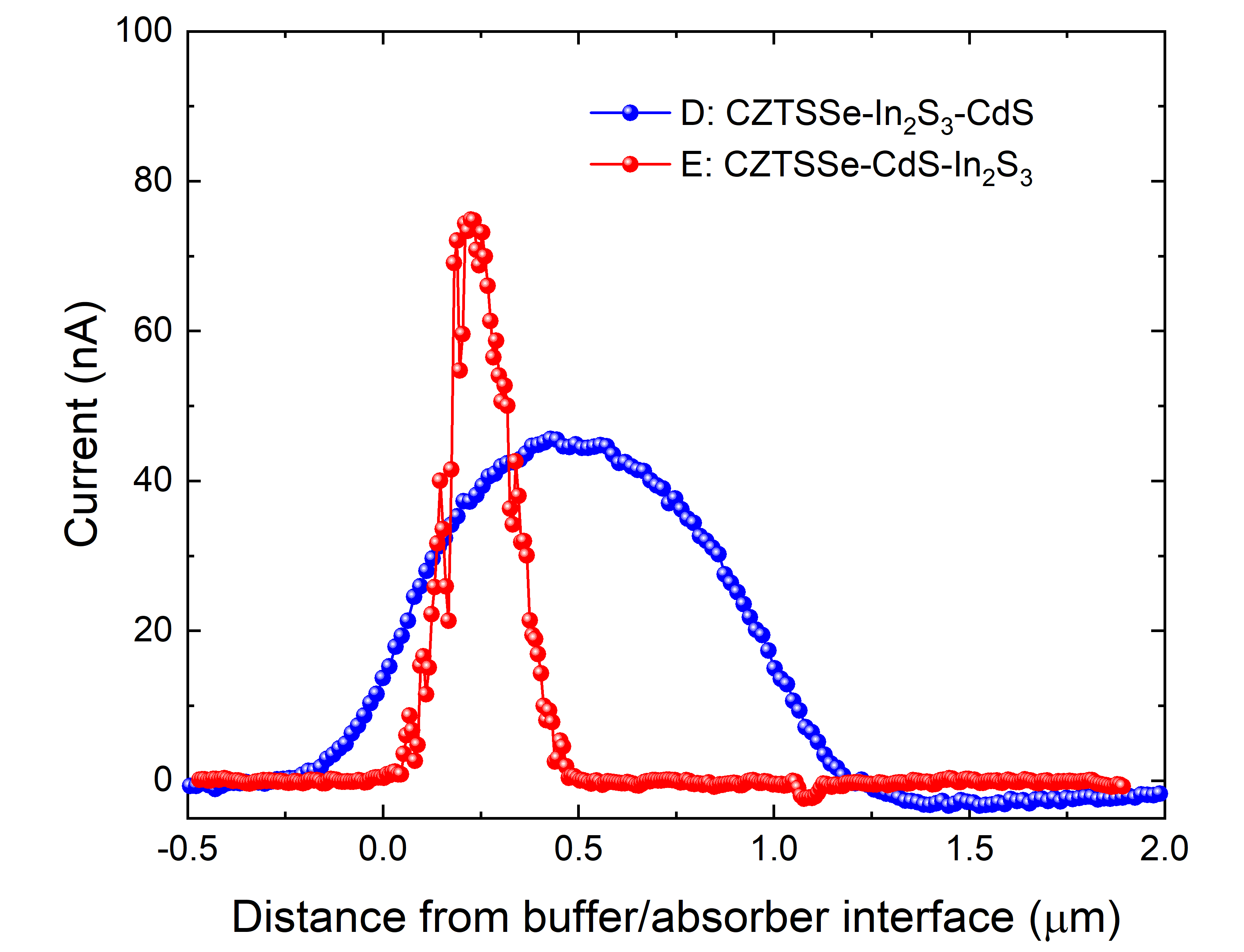

Supplement: Supplementary file 2 — ae3c01622_si_002.zip [file ae3c01622_si_002.zip › Raw_EBIC_dual buffer_CZTSSe_ comparison.png]

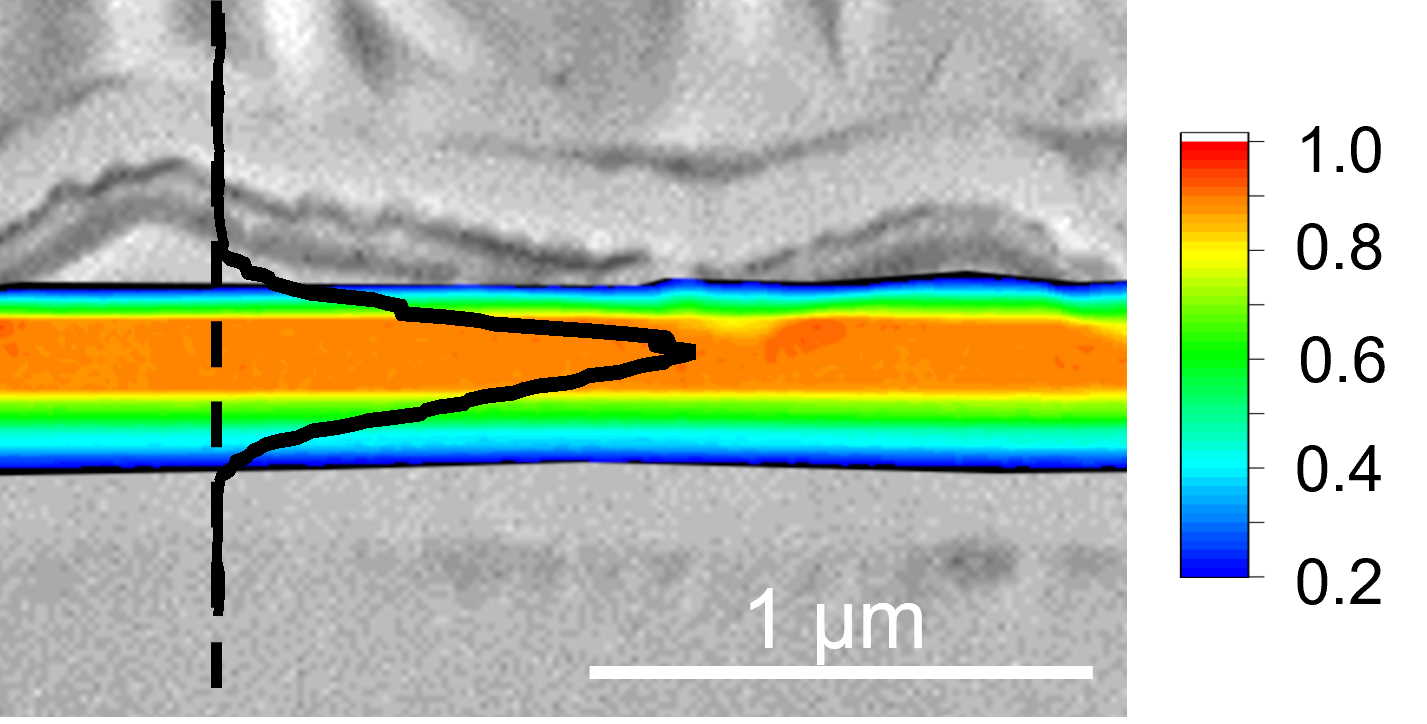

Supplement: Supplementary file 2 — ae3c01622_si_002.zip [file ae3c01622_si_002.zip › Graphical_abstract.png]

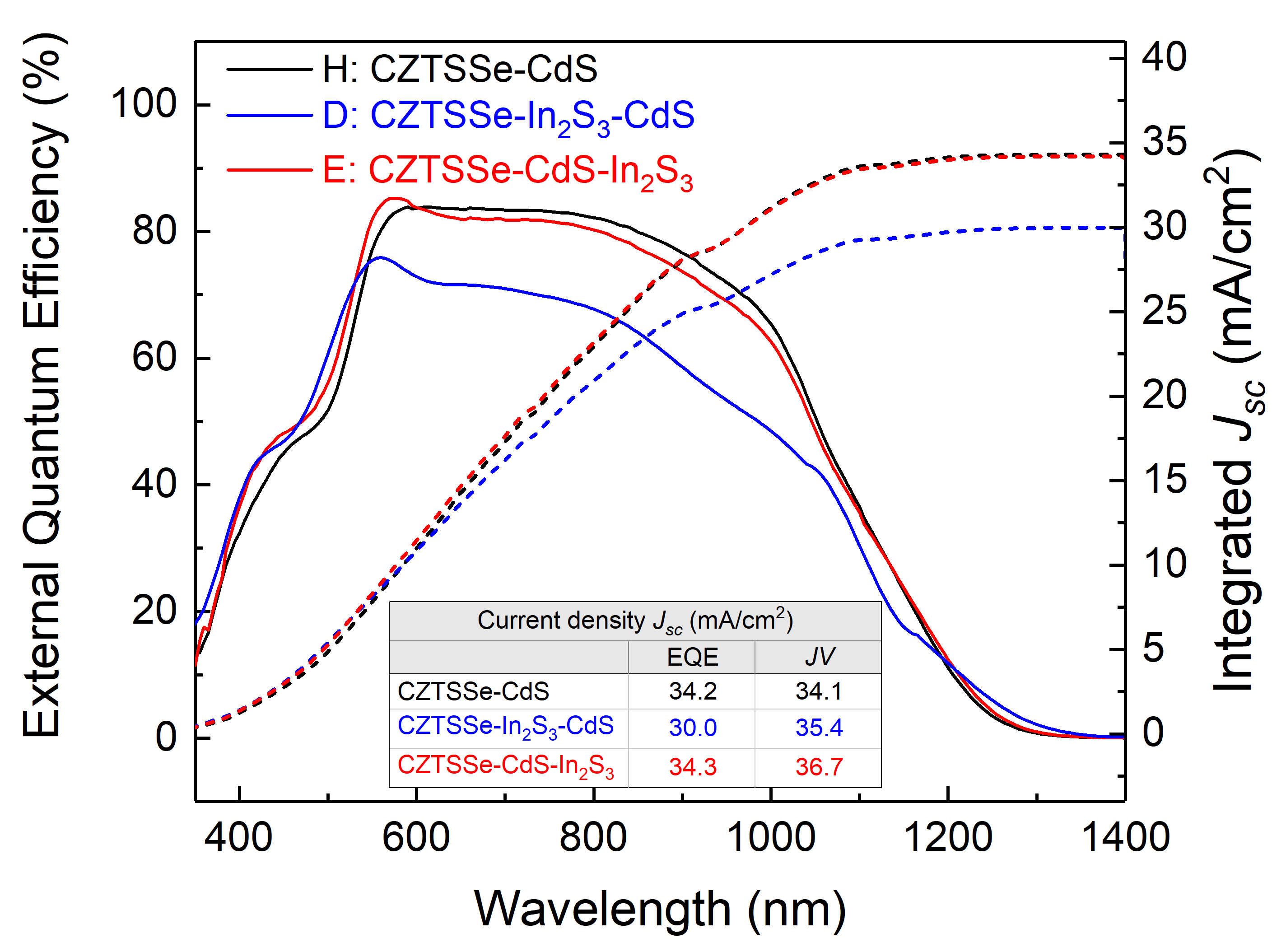

Supplement: Supplementary file 2 — ae3c01622_si_002.zip [file ae3c01622_si_002.zip › EQE_dual_buffers_CZTSSe_integrated_Jsc_revised.png]
